# Effect of Flavonoids on Oxidative Stress and Inflammation in Adults at Risk of Cardiovascular Disease: A Systematic Review

**DOI:** 10.3390/healthcare4030069

**Published:** 2016-09-14

**Authors:** Jenni Suen, Jolene Thomas, Amelia Kranz, Simon Vun, Michelle Miller

**Affiliations:** 1 Nutrition and Dietetics, School of Health Sciences, Flinders University, GPO Box 2100, Adelaide 5001, Australia; jenni.suen@flinders.edu.au (J.S.); jm.thomas@flinders.edu.au (J.T.); amelia.kranz@flinders.edu.au (A.K.); 2 Department of Vascular Surgery, School of Medicine, Flinders University, GPO Box 2100, Adelaide 5001, Australia; drsvun@gmail.com

**Keywords:** polyphenol, antioxidant, dietary intervention, prevention, cocoa, olive oil

## Abstract

Oxidative stress (OS) and inflammatory processes initiate the first stage of cardiovascular disease (CVD). Flavonoid consumption has been related to significantly improved flow-mediated dilation and blood pressure. Antioxidant and anti-inflammatory mechanisms are thought to be involved. The effect of flavonoids on markers of oxidative stress and inflammation, in at risk individuals is yet to be reviewed. Systematic literature searches were conducted in MEDLINE, Cochrane Library, CINAHL and SCOPUS databases. Randomised controlled trials in a Western country providing a food-based flavonoid intervention to participants with one or two modifiable risk factors for CVD measuring a marker of OS and/or inflammation, were included. Reference lists were hand-searched. The Cochrane Collaboration Risk of Bias Tool was used to assess study quality. The search strategy retrieved 1248 articles. Nineteen articles meeting the inclusion criteria were reviewed. Eight studies were considered at low risk of bias. Cocoa flavonoids provided to Type 2 diabetics and olive oil flavonoids to mildly-hypertensive women reduced OS and inflammation. Other food sources had weaker effects. No consistent effect on OS and inflammation across patients with varied CVD risk factors was observed. Study heterogeneity posed a challenge for inter-study comparisons. Rigorously designed studies will assist in determining the effectiveness of flavonoid interventions for reducing OS and inflammation in patients at risk of CVD.

## 1. Introduction

**Figure 1:**
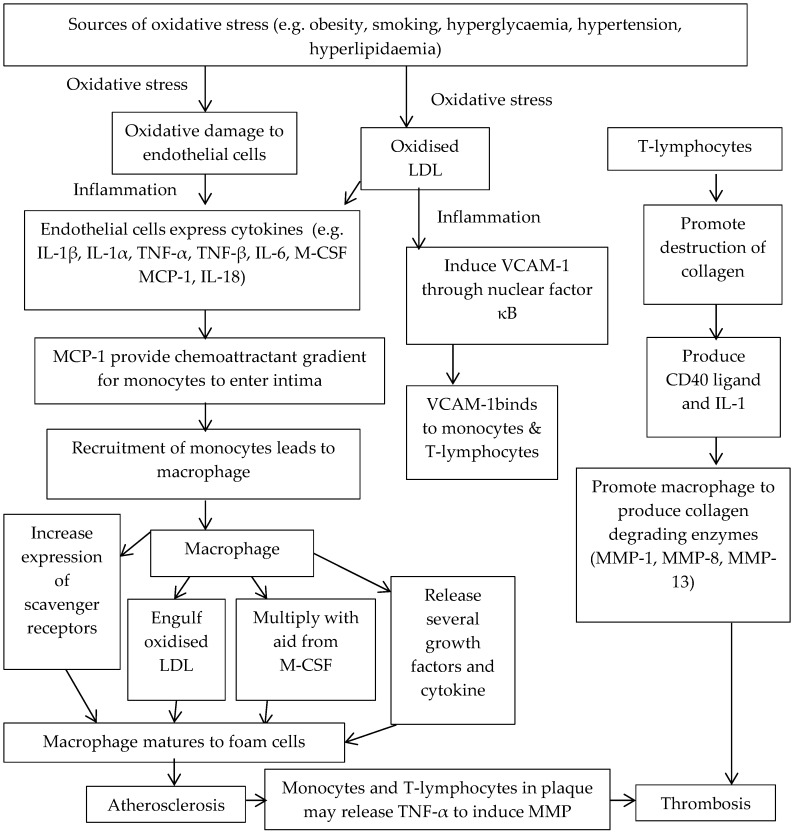
Atherosclerosis pathway involving oxidative stress (OS) and inflammation [4,5]. Abbreviations: LDL—low density lipoprotein. IL-1β—interleukin-1beta. IL-1α—interleukin-1alpha. TNF-α—tumour necrosis factor-alpha. TNF-β—tumour necrosis factor-beta. IL-6—interleukin-6. M-CSF—macrophage colony stimulating factor. MCP-1—monocyte chemoattractant protein-1. IL-18—interleukin-18. MMP—matrix metalloproteinases. VCAM-1—vascular cell adhesion molecule-1. IL-1—interleukin-1. MMP-1—matrix metalloproteinase-1. MMP-8—matrix metalloproteinase-8. MMP-13—matrix metalloproteinase-13.

Cardiovascular disease (CVD) is a major cause of death in Western countries such as Australia, America and countries within the European Union [1,2,3]. Oxidative damage and inflammatory processes initiate the first stage of CVD, characterised as atherosclerosis [4,5,6]. Oxidative damage results from the interaction of excess reactive free-radicals with cell structures [4]. Free radicals, normally produced by cells, enable communication between cells but enzymatic and non-enzymatic antioxidant mechanisms prevent harmful free-radical reaction with cell structures [4,6]. However, pathologic free-radical production is amplified by modifiable risk factors such as obesity, smoking or diabetes [4]. Excess free radical production leads to endothelial damage enabling oxidised low-density lipoprotein (OxLDL) to initiate the inflammatory process by stimulation of atherosclerotic mediators leading to atherosclerosis [4,5]. Figure 1 details the oxidative stress (OS) and inflammatory processes involved in the atherosclerosis pathway.

Changes in diet, physical activity and smoking habits are widely documented interventions to successfully reduce the risk of CVD in at risk individuals [7]. The use of flavonoids, a subclass of polyphenols [8] as a new dietary intervention has gained mounting interest over the past 20 years. Evidence that flavonoids potentially exhibit antioxidant and anti-inflammatory properties through obstructing oxidative stress and suppressing pro-inflammatory mediators [9,10,11] and their abundance in foods associated with the Mediterranean diet has led this emergent interest [11].

A secondary analysis of the Zutphen Elderly Study was the first to provide epidemiological evidence that a higher intake compared to lower intake of flavonoids was associated with a lower risk of coronary heart disease (CHD) mortality (RR: 0.42, 95% CI: 0.20–0.88) [12]. A meta-analysis of healthy, at risk and individuals with established CHD noted that no randomised controlled studies had measured CVD morbidity or mortality as an outcome [13]. Therefore, the impact of flavonoids on CVD events is unknown. Alternatively, quantitative proxy parameters known to increase CVD risk such as high blood pressure (BP) or reduced flow mediated dilation (FMD) were measured [13,14]. Flavonoid consumption was associated with significant improvements in acute and chronic FMD and reduced systolic and diastolic BP [14]. Literature on the relationship between flavonoid consumption, effect on CVD risk factors, mortality and morbidity would further clarify the impact of flavonoids on CVD.

Nevertheless, the mechanism by which flavonoids were able to improve FMD and BP is unclear [13,14]. Limited studies have measured FMD or BP with markers of OS and inflammation. Therefore, it is unknown if antioxidant and anti-inflammatory processes are associated with the increase in FMD and reduction in BP observed. The effects of flavonoids may vary according to the presence of a modifiable CVD risk factor versus the presence of established heart disease. Therefore, if flavonoids exhibit antioxidant or anti-inflammatory action, their impact may be greater prior to established cardiovascular disease. That is, during atherogenesis, where oxidative stress and inflammation have the potential to initiate CVD. No systematic review has investigated the effect of flavonoids on individuals with CVD risk factors.

This systematic review aims to investigate the association between different flavonoid food sources and their effect on OS and inflammation in adults with one or two modifiable risk factors for CVD. As a secondary outcome, the flavonoid dosage in food sources investigated will also be compared to determine if any results observed are related to polyphenol or flavonoid dose.

## 2. Materials and Methods

### 2.1. Study Criteria

Randomised controlled trials investigating the effect of a flavonoid food intervention on a marker of oxidative stress and/or inflammation in participants with one or two modifiable risk factors for CVD are included in this review. Interventions were additionally required to report flavonoid or polyphenol content as a numerical value to enable comparisons between interventions.

### 2.2. Search Strategy

MEDLINE, Cochrane Library, CINAHL and SCOPUS were used to perform the search that was carried out on 6 September 2014. Both MeSH terms and keywords variations were used and articles limited to “Human” and “English language” where possible. Search terms applied to the search strategy were sought under “Title, Abstract, Keywords” and under “All Text” when “Title, Abstract, Keywords” was not provided as a combined option. No other limits were applied to the search strategy. The search strategy intended to yield flavonoid intervention articles measuring oxidative stress or inflammation in adults. Search terms used to identify flavonoid foods included: polyphenol or flavonoids or anthrocyanin or catechin or flavon* or isoflavon* or benzoflavone or proanthrocyanidin. Oxidative stress or inflammat* was used to identify the outcome measure. Adult and Aged were the terms used to identify the target group.

Example search strategy applied to the Cochrane Library (Polyphenol or Flavonoids or Anthocyanin or Catechin or Flavon* or isoflavon* or benzoflavone or proanthrocyanidin) and (Oxidative stress or inflammat*) and (Adult or Aged).

### 2.3. Study Selection

**Figure 2:**
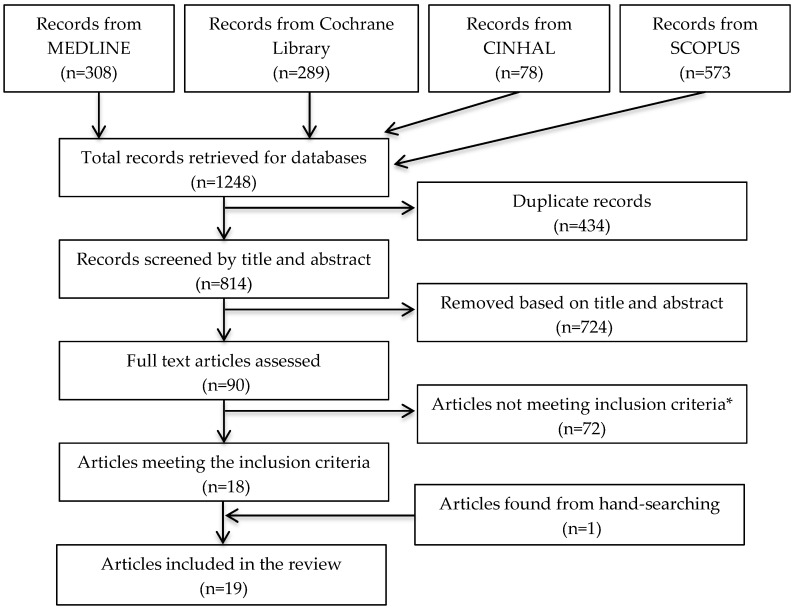
Flow diagram of study selection * Exclusions were in the following order for the following reasons: >2 modifiable risk factors for CVD (*n* = 37), Used supplementation (*n* = 31), Conducted in non-Western countries (*n* = 2), Data could not be separated for interpretation (*n* = 2).

The search strategy yielded a combined total of 1248 articles and 90 relevant articles to be assessed against the secondary exclusion criteria detailed below. Figure 2 details the study selection process.

The provision of flavonoid through supplements was excluded, as the effect of flavonoids obtained from food alone was the topic of this review. Supplements were defined as an extracted component of a flavonoid rich food (e.g., extracts or flavonoid containing capsules). Foods that were freeze-dried and administered in powder form were not considered a supplement as they contained the content of the whole food within its food matrix rather than solely an extracted component. Only studies conducted in Western countries were included on the basis of lower incidence of CHD in many Asian countries compared to Western countries, due to differences in food supply and the background diet that would likely be higher in usual polyphenol intake than the background diet of Western populations and therefore excluded to reduce sources of confounding.

Studies were hand searched to ensure that adults had one or two modifiable risk factor/s. Risk factors of interest included Type 2 diabetes, hypertension, high cholesterol, smoking, dyslipidaemia, obesity and overweight. Studies with less than three modifiable risk factors were included to exclude participants with metabolic syndrome that theoretically would be associated with higher levels of oxidative stress and inflammation that would be incomparable to those at a lower risk.

### 2.4. Data Extraction and Quality Assessment

A single author screened the articles and developed the results tables. Articles were screened for relevance against the selection criteria and categorised by their primary food. Result tables were created from extracted data. Risk factors for CVD, quantitative flavonoid intervention, and findings at baseline, post intervention and change observed were noted. Findings were reported as mean and standard deviations. Authors were not contacted for additional information.

### 2.5. Quality Assessment

Study design quality was assessed using The Cochrane Collaboration’s Risk-of-bias tool with guidance of the Cochrane Collaboration Handbook of Systematic Reviews of Interventions [15]. Briefly, the tool consisted of six domains assessing the risk of selection, performance, detection, attrition, reporting and other bias. Other biases are suggested to be specific to the study question. Therefore, particular attention was paid to intention-to-treat-analysis in parallel studies and washout periods between interventions in crossover studies. Confounders (e.g., background diet, physical activity, and adherence) and presence or absence of a method to monitor side effects were also noted when considering strengths and limitations of study design. Two blinded authors independently assessed each article’s source of bias as high-risk, unclear-risk or low-risk of bias using quotes from the article to support their judgment. Following the assessment of all studies, authors reported their respective results and discussed any difference to reach the final conclusion. If a final conclusion was unable to be achieved, a third author was required to determine the final conclusion.

### 2.6. Interpreting Outcome Measures

It is well documented that oxidative stress and inflammation is associated with disease. Therefore, any reductions in markers that mediate oxidative stress measured in urine, plasma or platelets were deemed as evidential findings suggestive of antioxidant activity. Such oxidative markers included urinary 15-F2-isoprotane, urinary 8-hydroxydeoxyguanosine (8-OHdG), urinary F2 isoprostane, plasma 8-isoprostane, platelet 8-isoprostane or oxLDL.

An increase in any mediator related to the presence of antioxidant activity was interpreted as positive findings for the purpose of this review. These mediators included: malondialdehyde, carboxyl groups, ferric reducing/antioxidant power (FRAP), oxygen radical absorbance capacity (ORAC), free radical-scavenging capacity (FRSC), total antioxidant capacity (TAC) and glutathione. 

In contrast to the number of mediators related to the presence of antioxidant activity, clinically meaningful improvement in OS remains uncertain in current literature. The ability to accurately measure OS markers consistently has been a limitation affecting its evolvement as a clinical outcome measure [16]. Currently no marker of OS has been associated with the relative risk of developing CVD [16]. Therefore, the ability of flavonoids to clinically improve oxidative stress or relative risk of developing CVD could not be assessed.

Similarly, a reduction in markers associated with the inflammatory process was assessed as suggestive findings that flavonoids reduce inflammation.

Vascular cell adhesion protein 1 (VCAM-1) and intercellular adhesion molecule 1 (ICAM-1) are examples of inflammatory markers. High sensitive c-reactive protein (hs-CRP), an inflammatory marker has been associated with the relative risk for developing CVD [17] where high relative risk is defined as >3.0 mg/L, average relative risk as 1.0 to 3.0 mg/L and low relative risk as <1 mg/L. Studies measuring hs-CRP were assessed for their ability to reduce relative risk of CVD via these guidelines. No guidelines were made for the use of ICAM-1 and VCAM-1 as there are no World Health Organisation Standards available, markers are unstable unless frozen making it difficult for research purposes and assays available for testing are limited [17].

## 3. Results

The search strategy yielded 1248 articles. Eighteen articles remained after the removal of duplicates and assessment against inclusion criterions. One article was found through hand-searching reference lists. A total of nineteen articles were included (Figure 2) consisting of six parallel randomised controlled trials and thirteen crossover randomised controlled trials. The exclusion of non-Western countries only removed two studies, both performed in Japan.

The number of participants in each study ranged from *n* = 10–133. A considerable degree of heterogeneity existed across all studies for characteristics such as the flavonoid foods administered, the gender included, their risk factor(s) for CVD and whether OS and/or inflammation was investigated (Table 1).

Eight studies were assessed to have overall low risk of bias and eleven at an overall unclear risk of bias (Table 2). Main sources of bias related to selection, performance, attrition and other sources of bias. Detection bias was generally low as all outcome measures were objective measures. In all cases, the investigator performing data analysis was blinded and in one case where outcome data analysis was conducted separately by a statistician [18]. Seventeen studies were assessed as low risk of reporting bias, one study at an unclear risk as the outcome measures were not well defined [19] and one at high risk because a primary outcome measure was not reported [18].

The main types of foods containing flavonoids were fruits, cocoa, vegetables, modified grain foods, olive oil, tea and red wine. The number of studies that explored each main type of flavonoid containing food is displayed in Table 1. Results will be discussed according to these sources of flavonoids.

### 3.1. Cocoa

Table 3 and Table 4 summarise the study methodology and findings for four studies, which administered flavonoids in the form of cocoa. Of the four studies, two reported statistically significant improvements in a marker of OS or inflammation compared to baseline. Mellor et al. demonstrated that a 121.5 mg dose of flavonoids through cocoa powder led to an increase in all OS and inflammatory markers measured while a higher dose (472.5 mg) trended towards a reduction in all OS and inflammatory markers in type 2 diabetics [20]. Similarly Sarria et al. also observed a trend of reduced markers of OS and inflammation with 417 mg of flavonoids as cocoa powder in skim milk, led to a significant reduction in IL-10 and IL-1β when administered to adults with hypercholesterolaemia [21]. No significant reductions in OS markers and other inflammatory markers were observed [21]. Carnevale et al. was also able to demonstrate a significant reduction in OS when dark chocolate was administered to smokers as a flavonoid source as opposed to milk chocolate [22].

Considering studies conducted with smokers [22] and participants with type 2 diabetes [20,23] were assessed as at low risk of bias, these finding suggest that cocoa polyphenols could improve OS and inflammation. 

Sarria and co-workers’ study on hypercholesterolaemic adults was considered as at unclear risk of bias. A washout period between intervention crossover and control treatments was not initiated [21]. This may have caused effects of the intervention or control to be carried over to the second phase, potentially leading to results that are confounded. For this reason, it cannot be concluded that flavonoids exhibit antioxidant or anti-inflammatory properties in adults with hypercholesterolaemia.

### 3.2. Fruit

Six studies administered fruit polyphenols in various forms such as whole fruit (*n* = 1), fruit juice (*n* = 1) or freeze-dried fruit powder (*n* = 4). Table 5, Table 6, Table 7 and Table 8 summarise the methodology and findings from each study.

Mixed results and quality of study design led to inconclusive findings. Three studies observed significant findings while the remaining three observed no significant findings.

Burton-Freeman et al. observed a significant reduction in OxLDL levels (Δ OxLDL: −7.3, *p* = 0.0008) six hours after the ingestion of a strawberry beverage containing 338 mg flavonoids with a high fat meal in 24 overweight participants with hyperlipidaemia [24] . In contrast, no significant difference was observed in OS and inflammation when Basu et al. provided high and low doses of freeze-dried strawberry to participants with the same risk factor over 12 weeks [25]. No significant differences were observed between or within groups when Auclair et al. provided two different doses (0.21 g or 1.43 g) of apple flavonoids to 10 males with hypercholesterolaemia (Table 5 and Table 6) [26].

A study by Rankin et al. used raisins as a source of polyphenols in a placebo-controlled trial of 17 obese adults [27]. When comparing the groups, there were no statistically significant differences in markers of OS or inflammation, however the reduction in OS (urinary 8-epiPGF2α: −1056.5 vs. −813.2 pg/mg creatinine) and improvement in antioxidant activity (ORAC: +451 vs. +67.4 μmol/L TE) was greater in the intervention group compared to baseline [27].

Two studies found mixed changes in inflammatory markers. The consumption of 94.66 mg polyphenols in a strawberry beverage given to 24 overweight individuals along with a high-carbohydrate moderate-fat meal compared to placebo led to significantly lower levels of hs-CRP (2.7 mg/L vs. 3.1 mg/L, *p* = 0.02) and IL-6 (2.6 ng/L vs. 3.1 ng/L, *p* = 0.05) [28]. Lowered hs-CRP reduced relative risk of CVD from high risk to average risk. No changes in IL-1β and TNF-α were observed (Table 8). Similarly, the administration of 500 mL/day of high flavonoid dose cranberry juice compared to low flavonoid dose cranberry juice in 23 overweight men led to a significant between group reduction in ICAM-1 (−11.5 vs. 14.4 ng/mL, *p* < 0.05) [29]. However, no significant effects were observed in other inflammatory markers (Table 5 and Table 7).

Four studies were considered to be at unclear risk of bias and two studies to be at low risk of bias. Methods of reducing selection bias were consistently poorly reported across all studies. All studies randomised participants into their respective groups, however the method of randomisation was not reported and no studies reported on allocation concealment. Although Rankin et al. [27] and Burton-Freeman et al. [24] had positive findings the study quality was assessed as unclear risk of bias as no washout period between interventions was provided. The data provided in the second intervention period would be at risk of inflation leading to an overall overstated outcome. The investigators were also unmasked from the intervention provided which carries a degree of detection bias. Nevertheless, potential confounders were considered suggesting that the positive effect observed is more likely to be due a direct result of the intervention provided. In the two studies with mixed results, Edirisinghe et al. [28] and Ruel et al. [19] were considered to be at unclear risk of bias. Ruel et al. [19] did not consider sources of confounding. The reductions in inflammatory markers observed after the cranberry juice intervention may therefore not have been a result of this intervention. Investigators were unmasked and Edirisinghe et al. [28] did not report baseline inflammatory markers, suggesting a degree of detection and reporting bias.

Auclair et al. [26] and Basu et al. [25] whom reported no significant findings were assessed as at a low of bias.

### 3.3. Vegetable

Only Wright et al. administered polyphenols using a vegetable source. This study assessed the effect of dried-purple carrot consumption over four weeks on inflammation in 16 overweight men [29]. At the end of the intervention, the percentage of individuals that had a CRP level ≤2 mmol/L increased by 12.5% and those with CRP level ≥3 mmol/L decreased by 12.5% [29]. However, these changes were not statistically significant [29]. Between group differences were not reported but did not appear to be clinically significant [29].

Wright et al. was considered to be at a low risk of bias as it was a double-blind study and accounted for confounders to ensure that any effects observed would be related to the intervention [29]. All participants were also accounted for during data analysis and outcome data was reported accordingly. The methods of randomisation and allocation concealment were not reported. A sample size calculation was also not reported.

Although this study reported considerable efforts to reduce the risk of bias, the results of one study that could be underpowered cannot provide conclusive findings and hence is unable to inform clinical practice until further research has been conducted.

### 3.4. Modified Grain Foods

Clerici et al. and Yang et al. administered flavonoids via a grain-based food (i.e., pasta or bread) modified with added soy [30] or lupin [31]. Both studies compared the modified grain food with flavonoid doses ranging from 31 to 173 mg to a non-modified grain equivalent. Overweight, obesity or Type 2 Diabetes were the CVD risk factors present in these participants. Table 9 summaries the methodology and finding of these studies.

Clerici et al. observed that the consumption of 31–33 mg isoflavones was associated with a significant reduction in OS (Δ OxLDL: −1.60 U/mL, *p* = 0.009, Δ8iso-PGF2α: −123.8 pg/mL, *p* = 0.001) and an increase in antioxidant activity (ΔTAC: 79 mmol/L, *p* = 0.0002, Δ Glutathione: 5.0 mmol/L, *p* = 0.0003) when compared to the group administered the unmodified grain food [30]. Changes in IL-6 trended toward significance between the groups (+0.19 vs. +0.98 pg/mL, *p* = 0.087) [30]. In contrast to those reported by Yang et al. who provided lupin-fortified bread containing 173 mg of flavonoids to 74 overweight and obese adults. They observed no significant differences in OS (plasmaF2-isoprotane: 45 pmol/L, 95% CI: −68, 158, urinary F2-isoprostanes: 17 pmol/mmol creatinine, 95% CI: −43, 76) between the intervention and control groups and no significant within group changes (Table 9) [31].

Both studies were considered to have an unclear risk of bias. Clerici et al. conducted a double-blind study, however methods of randomisation and allocation concealment were not reported and the study did not consider sources of confounding. It therefore cannot be concluded that the findings are a result of the intervention. However, potential carry-over effects were accounted for and all outcome measure data was reported. Yang et al. did not achieve the sample size required to see a change in the outcome measured as predicted suggesting a risk of type 1 error. Therefore, the ability of flavonoids provided from soy and lupin added to grain food to affects OS and inflammation in individuals with cardiovascular risk factors remains inconclusive.

### 3.5. Olive Oil

Table 10 summarises the methodology and findings of the olive oil flavonoid intervention studies. This intervention was compared with a placebo-oil or a lower dose of polyphenols or oil supplemented with epigallocatechin 3-gallate (EGCG). Dose of olive oil polyphenols ranged from 3.20 mg to 30 mg/day.

The provision of olive oil flavonoid compared to placebo by Moreno Luna et al, significantly reduced OS (*p* < 0.01) and inflammation (*p* < 0.001) [32]. While Ruano et al. observed a larger reduction in OS (*p* < 0.001) when 15.98 mg of olive oil flavonoids were consumed compared to a lower dose olive oil polyphenols in hypercholesterolaemic individuals (Table 10) [33].

High sensitivity-CRP was measured in Moreno-Luna et al. where the provision of olive oil polyphenols to mildly-hypertensive-young women was associated with a −1.3 mg/L reduction [32]. The average hs-CRP was 1.6 mg/L at baseline, which renders the participants at an average risk of developing CVD and the reduction observed would theoretically bring the participants to a low risk defined as <1 mg/L [17,32]. However, the potential for other components within the olive oil food matrix to be responsible for the effects observed cannot be excluded. The study methodology does not suggest that any side effects were monitored, although three participants who dropped out reported intolerance to the oil [32].

In individuals with endothelial dysfunction the addition of 30 mL/day of uncooked olive oil providing 10.2 mg of flavonoids significantly reduced the inflammation marker ICAM-1 (Δ: −9 pg/nL, *p* < 0.001) [18]. Insignificant reductions in VCAM-1, IL-6 and hs-CRP were observed (Table 10) [18]. Whist an increase in OS (ΔOxLDL + 0.02, *p* = 0.79, Δ plasma-isoprostane (Δ: 34, *p* = 0.003) was observed [18], no significant differences were reported when compared to the EGCG olive oil supplemented group [18]. Baseline and post-intervention data on the supplemented group were not reported.

These findings suggest that the provision of olive oil polyphenols led to a reduction in OS in hypercholesterolaemic and hypertensive individuals. However, the effect observed in hypercholesterolaemic individuals should be interpreted with caution as this study was assessed as an unclear risk of bias. Although all outcome measures were reported in this study, the method of randomisation, allocation concealment and presence or absence of dropouts was poorly reported. The main sources of bias from this study were a result of not accounting for potential carry-over effects and participants and investigators being unmasked which could lead to an overstated result. Nevertheless the study conducted with hypertensive individuals was considered to be at low risk of bias as it was a double-blind study where confounding was considered and randomised sequence generation and a washout period were reported.

A reduction in inflammatory markers but not OS in individuals with endothelial dysfunction was suggested to be a result of the fat content provided [18]. However, this study was also considered to be at an unclear risk of bias because methods of randomisation and allocation concealment were not reported and changes in diet and exercise were not considered. Although not stated, the results suggest that an intention-to-treat analysis was conducted. Nevertheless, the study ensured that the statistician conducting the data analysis was masked and monitored participant adherence.

### 3.6. Tea

Two parallel studies investigated the effects of black tea or green tea on OS and inflammation in smokers. The study details are described in Table 11. DeMaat et al. compared the consumption of black tea against green tea or green tea isolate on inflammatory markers while Hakim et al. compared decaffeinated green tea, decaffeinated black tea and water on OS markers [34,35].

No significant differences in inflammatory markers were observed when water, black tea, green tea or green tea isolate was administered to 64 healthy smokers for four weeks [34]. Similarly, when decaffeinated black or green tea or water was provided for four months, changes in urinary 8-OHdG were also insignificant [35]. Neither study found significant within group changes in markers of OS and inflammation (Table 11).

DeMaat et al. was considered to be at an unclear risk of bias as investigators were unmasked from the intervention and although participants were masked, methods of masking were not reported. Randomisation and allocation concealment was also poorly reported. Similarly, Hakim et al., which was considered to be at a low risk of bias, also did not report allocation concealment. However, the multiple analyses of urinary 8-OHdG were performed in this study reducing measurement bias and random sequence generation was also reported. Both studies also accounted for confounders. Regardless of the study quality, the effect of tea polyphenols on OS and inflammation in smokers remains inconclusive.

### 3.7. Wine

Abu-Amsha Cacetta et al. compared the effects of dealcoholised red wine, red wine and white wine on markers of OS in 18 smokers [36]. No significant changes were observed after two weeks of daily intervention compared to baseline (Table 12). However, in comparison to red wine and white wine, dealcoholized red wine significantly reduced OS (Δ plasma F2-isoprostane: −179.1 pmol/L vs. +1.1 pmol/L and −35.8 pmol/L, *p* ≤ 0.05) [36]. These finding suggest that the alcohol content of the wine may reduce the bioavailability of flavonoids.

This finding should be interpreted with caution as this study is considered to be at an unclear risk of bias. The study used a random sequence generation technique to randomise participants but allocation concealment was not reported. Participants and investigators were unmasked and although all outcomes intended were reported, the results suggest that not all data values were included in data analysis. Therefore, the changes observed may not be due to the intervention and the effect of wine flavonoids on OS and inflammation in smokers remains inconclusive.

## 4. Summary

There does not appear to be any consistent effect of flavonoids on OS and inflammation across patients with varied CVD risk factors. From the low-risk-of-bias studies with positive findings, the provision of cocoa polyphenols to type 2 diabetics [20] and olive-oil polyphenols to mildly-hypertensive women appear to improve OS and inflammation [32]. Cocoa also appears to improve oxidative stress in smokers [22]. However, side effects associated with the interventions were not monitored across all studies and may not be monitored due to the abundance of polyphenols in everyday foods.

Aside from the methodological flaws across all studies (Table 1), within group reductions in OS were observed when cocoa was provided individuals with hyperlipidaemia [21]. A significant within group reduction in OS was also observed when soy fortified bread was provided to participants with type 2 diabetes [30]. Dealcoholised red wine also significantly reduced OS in smokers [36]. No significant between or within group reduction in OS and inflammation were observed with other food sources. Insignificant reductions in OS and inflammation were observed when olive oil polyphenols were provided to individuals with endothelial dysfunction [18].

Therefore, cocoa and olive oil polyphenols appear to have the greatest potential to reduce OS and inflammation. Soy fortified bread and dealcoholised red wine also appear to improve markers of OS. Fruit and vegetable sources of flavonoids are inconclusive; studies with greater sample sizes may prove more informative. 

### Strengths and Limitations

To the authors’ knowledge, this is the first review to investigate the effect of polyphenols on oxidative stress and inflammatory markers in adults with CVD risk factors. No date limits were applied when the search was conducted and only included randomised controlled trials. Therefore, the source of information used was best to address this review question. Two reviewers independently assessed study quality using an establish critique tool and the guidance from the tool’s author, reduced the bias associated with subjective assessment of study quality.

However, study heterogeneity was the major limitation to provide conclusive findings. The risk factor/s for CVD, type of polyphenol intervention, intervention duration, marker of oxidative stress and/or inflammation measured varied amongst the studies. Some studies reported on the complete polyphenol content provided by the intervention per day while other only reported on the polyphenolic subclass of interest per day (e.g., isoflavone, and flavonoid). This negatively impacted on the ability of this review to report any findings associated with dose.

The study quality as determined by The Cochrane Collaboration Risk of Bias Tool further limited the results to be weighed upon single studies. An assumption was also made that a reduction in the oxidative stress markers or increase in antioxidant activity markers observed would be findings that the intervention reduced oxidative stress. The assumption that a reduction in inflammatory markers meant a reduction in inflammation was also made when interpreting the results from inflammatory markers. There is reason to be cautious in this assumption as the absorption and retention of exogenous antioxidants is under debate [37].

## 5. Conclusions

These results are drawn from single studies of varied polyphenol food sources on individuals with modifiable risk factors for CVD. It cannot be concluded if a particular food source is more superior to another, nor whether the use of flavonoids is more appropriate in one particular modifiable risk factor of CVD over another. The avoidance of other food sources high in polyphenols during the study period and side effects not being monitored and consistency in reporting the intervention as polyphenol content or solely a polyphenol subclass of interest also limit the applicability of these results to practice. However, these foods are regularly abundant in the diet and appear to do no harm in moderate doses.

Therefore, although cocoa and olive oil polyphenols appear to have the potential to reduce OS and inflammation in adults with ≤2 CVD risk factors, further research in this area is required.

### 5.1. Implications for Practice

The ability of flavonoids to reduce oxidative stress and inflammation appear promising, however results are too premature to incorporate in to clinical practice or to inform the development of health claims regarding flavonoids and flavonoid-containing foods.

### 5.2. Implications for Research

Future research should be conducted across all sources of polyphenols in individuals with a modifiable risk factor for CVD as there are limited studies conducted in the area and methodological flaws in most existing studies. 

Although flavonoid supplementation was not a direct topic of this review, this review did highlight the abundance of flavonoid supplementation studies. Recently, research such as Davinelli et al. [38] suggests that a review on this topic could also add to this area. Further research on gut microbiota and its potential for flavonoid action or absorption could further strength the debate on flavonoid bioavailability. Promising work on polyphenols is also occurring in successful aging [39] and exploration of the bioactive compounds in fruit [40].

Total polyphenol content of the intervention as well as flavonoid content should also be reported in future studies to provide clarification whether flavonoids are more bioactive than other classes of polyphenols in adults with a modifiable CVD risk factor/s. If there are beneficial findings, systematically reporting polyphenol content and flavonoid content will also enable further studies to define the dose required to achieve clinical outcomes, increasing the applicability for practice recommendations. 

More rigorous randomised placebo-controlled trials with large samples should be conducted including both women and men. To enable comparisons between changes in OS and inflammation, future studies should consider measuring all markers of OS and inflammation especially hs-CRP as it is a widely accepted marker of with CVD risk.

## Figures and Tables

**Table 1:** Heterogeneity of studies meeting studies meeting the inclusion criteria.

Study Characteristics	Number of Studies
**Flavonoid food**	
Cocoa/chocolate	4
Fruit/fruit juice	6
Vegetable	1
Modified grain food	2
Oil	3
Tea	2
Red wine	1
**Gender**	
Male and Female	14
Male	4
Female	1
**Risk factors**	
Smoking	4
Overweight	3
Obesity	1
Overweight/obesity	1
Hypertension	1
Hyperlipidaemia	5
Type 2 Diabetes	3
Endothelial dysfunction	1
**Measured markers of**	
Oxidative stress only	6
Inflammation only	4
Oxidative stress and inflammation	9

**Table 2:** Quality critique of trials with Cochrane Collaboration Tool for assessing risk of bias [15].

Author, Year	Selection Bias	Performance Bias	Detection Bias	Attrition Bias	Reporting Bias	Other Bias	Overall Risk of Bias
	Random Sequence Generation and Allocation Concealment	Blinding or Participants and Personnel	Blinding Outcome Assessment	Incomplete Outcome Data	Selective Reporting	Other Sources of Bias (e.g., Carry Over Effect, Confounding, Adherence to Intervention)	
Mellor, 2013 [20]	Unclear	Low	Low	Low	Low	Low	Low
Sarria, 2014 [21]	Unclear	Unclear	Low	Low	Low	Unclear	Unclear
Carnevale, 2012 [22]	Low	High	Low	Unclear	Low	Low	Low
Mellor, 2010 [23]	Unclear	Low	Low	Low	Low	Low	Low
Burton-Freeman, 2010 [24]	Unclear	Unclear	Unclear	Low	Low	High	Unclear
Basu, 2014 [25]	Unclear	Unclear	Low	Low	Low	Low	Low
Auclair, 2010 [26]	Unclear	Low	Low	Low	Low	Low	Low
Rankin, 2008 [27]	Unclear	Unclear	Low	Unclear	Low	Unclear	Unclear
Ruel, 2013 [19]	Unclear	Low	Low	Low	Unclear	Unclear	Unclear
Edirisinghe, 2011 [28]	Unclear	Low	Low	Low	Low	Unclear	Unclear
Wright, 2013 [29]	Unclear	Low	Low	Low	Low	Low	Low
Clerici, 2011 [30]	Unclear	Low	Low	Low	Low	Unclear	Unclear
Yang, 2010 [31]	Unclear	Unclear	Low	Unclear	Low	Unclear	Unclear
Moreno-Luna, 2012 [32]	Unclear	Unclear	Low	Unclear	Low	Low	Low
Ruano, 2005 [33]	Unclear	Unclear	Low	Unclear	Low	High	Unclear
Widmer, 2013 [18]	Unclear	Unclear	Low	Unclear	Unclear	Low	Unclear
deMaat, 2000 [34]	Unclear	Unclear	Low	Unclear	Low	Low	Unclear
Hakim, 2003 [35]	Unclear	High	Low	Unclear	Low	Low	Low
Abu-Amsha Caccetta, 2001 [36]	Unclear	Unclear	Low	Unclear	Low	Unclear	Unclear

**Table 3:** Effect of cocoa polyphenols on oxidative stress markers at baseline and post-intervention.

Author, Year	Risk Factor for CVD	Intervention/s	Marker Measured	Results			
				Baseline	Post-Intervention	Δ	*p*-Value
Mellor, 2013 [20]	Type 2 diabetes	472.5 mg cocoa polyphenol with water on one occasion	15-F2t-isoprotane (mg/mol)	117.7 ± 4.0	116.8 ± 5.7	−0.9	0.48
		121.5 mg cocoa polyphenol with water on one occasion	15-F2t-isoprotane (mg/mol)	110.4 ± 3.0	207.1 ± 5.7	+96.7	0.02
Sarria, 2014 [21]	Total cholesterol >2000 mg/L	417 mg soluble polyphenols with 400 mL skim milk for 4 wks	MDA (nmol/mL)	2.50 ± 0.14	2.35 ± 0.18	−0.15	NS
			Carbonyl (nmol CG/gprotein)	0.15 ± 0.08	0.15 ± 0.08	0	NS
			FRAP (μM TE)	568.4 ± 23.41	556 ± 25.8	−12	NS
			ORAC (μM TE)	15,150 ± 647	15,983 ± 612	+833	NS
			ABTS (μM TE)	3696 ± 69	3616 ± 77	−80	NS
		400 mL skim milk for 4 wks	MDA (nmol/mL)	2.50 ± 0.14	2.55 ± 0.15	+0.05	NS
			Carbonyl (nmol CG/gprotein)	0.15 ± 0.08	0.20 ± 0.09	+0.05	NS
			FRAP (μM TE)	568.4 ± 23.41	548 ± 24.4	−20.4	NS
			ORAC (μM TE)	15,150 ± 647	15,366 ± 756	+216	NS
			ABTS (μM TE)	3696 ± 69	3683 ± 68	−13	NS
Carnevale, 2012 [22]	Smoking	40 g dark chocolate (≥85% cocoa) once	Platelet 8-iso-PGF2α (pmol/L)	~430 *	~385 *	−45	<0.05
		40 g milk chocolate (≤35% cocoa) once	Platelet 8-iso-PGF2α (pmol/L)	~445 *	~440 *	−5	NS

* = Values estimated from graphs. Results provided as mean ± SEM. Abbreviations: wks, weeks; NS, not significant and values not provided; MDA, malondialdehyde; FRAP, ferric reducing ability of plasma; ORAC, oxygen radical absorbance capacity; ABTS, 2,2′azino-di[3-ethylbenzthiazoline sulphonate]; 8-iso-PGF2α, 8-iso-prostaglandin F2-alpha; TE, Trolox equivalents; CG/gprotein, carboxyl groups per gram.

**Table 4:** Effect of cocoa polyphenols on inflammatory markers at baseline and post-intervention.

Author, Year	Risk Factor for CVD	Intervention/s	Marker Measured	Results			
				Baseline	Post-Intervention	Δ	*p*-Value
Mellor, 2013 [20]	Type 2 diabetic	472.5 mg cocoa polyphenol with water on one occasion	ICAM-1 (ng/mL)	325.6 ± 9.0	310.0 ± 8.4	−15.6	0.20
			E-selectin (ng/mL)	111.3 ± 5.8	96.6 ± 5.6	−14.7	0.09
			P-selectin (ng/mL)	253.0 ± 14.8	235.0 ± 7.7	−18	0.62
		121.5 mg cocoa polyphenol with water on one occasion	ICAM-1 (ng/mL)	321.1 ± 7.6	373.6 ± 10.5	+52.5	0.04
			E-selectin (ng/mL)	94.4 ± 4.0	105.8 ± 3.5	+11.4	0.28
			P-selectin (ng/mL)	265 ± 15.2	268.5 ± 12.4	+3.5	0.92
Mellor, 2010 [23]	Type 2 diabetic	16.6 mg catechins for 8 weeks	hs-CRP(mmol/L)	3.0 ± 0.6	2.0 ± 0.4	−1.0	0.22
		2 mg catechins for 8 weeks	hs-CRP(mmol/L)	2.6 ± 0.7	2.4 ± 0.6	−0.2	0.72
Sarria, 2014 [21]	Total cholesterol >2000 mg/L	417 mg soluble polyphenols with 400 mL skim milk for 4 weeks	IL-1β (pg/mL)	2.80 ± 0.39	1.85 ± 0.21	−0.95	0.001
			IL-6 (pg/mL)	4.16 ± 0.63	3.58 ± 0.70	−0.58	NS
			TNF-α (pg/mL)	6.00 ± 0.78	5.78 ± 0.68	−0.22	NS
			IL-10 (pg/mL)	14.47 ± 2.19	7.88 ± 1.44	−6.59	0.001
			IL-8 (pg/mL)	3.08 ± 0.46	2.67 ± 0.7	−0.41	NS
			VCAM-1 (ng/mL)	192.8 ± 17.4	169.3 ± 17.1	−23.5	NS
			ICAM-1 (ng/mL)	74.8 ± 17.0	73.5 ± 18.1	−1.3	NS
			MCP-1 (pg/mL)	94.5 ± 6.58	94.3 ± 7.40	−0.2	NS
		400 mL skim milk for 4 weeks	IL-1β (pg/mL)	2.80 ± 0.39	2.47 ± 0.25	−0.33	NS
			IL-6 (pg/mL)	4.16 ± 0.63	3.89 ± 0.59	−0.27	NS
			TNF-α (pg/mL)	6.00 ± 0.78	7.63 ± 0.65	+1.63	NS
			IL-10 (pg/mL)	14.47 ± 2.19	11.00 ± 1.43	−3.47	NS
			IL-8 (pg/mL)	3.08 ± 0.46	3.41 ± 0.41	+0.33	NS
			VCAM-1 (ng/mL)	192.8 ± 17.4	177.0 ± 14.8	−15.8	NS
			ICAM-1 (ng/mL)	74.8 ± 17.0	67.8 ± 16.2	−7	NS
			MCP-1 (pg/mL)	94.5 ± 6.58	88.4 ± 7.17	−6.1	NS

Abbreviations: NS, not significant and values not provided; ICAM-1, intercellular adhesion molecule; hsCRP, high-sensitive C-Reactive Protein; IL-1β, interleukin 1-beta; IL-6, interleukin 6; TNF-α, tumour necrosis factor-alpha; IL-10, interleukin 10; IL-8, interleukin 8; VCAM-1, vascular cell adhesion molecule-1; ICAM-1, intercellular adhesion molecule; MCP-1, monocyte chemoattractant protein-1.

**Table 5:** Effect of fruit polyphenols oxidative stress at baseline and post-intervention.

Author, Year	Risk Factors for CVD	Intervention/s	Marker Measured	Results			
				Baseline (Mean ± SD)	Post Intervention	*p*-Value	Δ
Auclair, 2010 [26]	High cholesterol	40 g/d Freeze-dried golden delicious apple powder (0.21 g/d polyphenols) + ½ glass of water for 4 weeks	FRAP (µM Fe^2+^/mL)	1047 ± 125	1021 ± 121	NS	−26
			ORAC (103 µmol TE/L)	14.1 ± 2.8	13.5 ± 2.5	NS	−0.6
		40 g/d of Freeze-dried cider apple powder (1.43 g/d polyphenols) with ½ glass of water for 4 weeks	FRAP (µM Fe^2+^/mL)	1026 ± 102	1057 ± 147	NS	+31
			ORAC (10^3^ µmol TE/L)	14.1 ± 2.7	13.4 ± 2.4	NS	−0.7
Basu, 2014 [25]	Obese and elevated serum lipids	25 g/day calorie and fibre matched control with 474 mL water for 12 weeks	MDA and HNE (µmol/L)	2.3 ± 2.3	2.1 ± 1.5	NS	−0.2
		25 g/day freeze-dried strawberry (1.08 g/d flavonoids) with 474 mL water for 12 weeks	MDA and HNE (µmol/L)	1.9 ± 2.3	1.3 ± 1.5	NS	−0.3
		50 g/day calorie and fibre matched control with 474 mL water for 12 weeks	MDA and HNE (µmol/L)	2.4 ± 2.3	2.3 ± 1.5	NS	−0.1
		50 g/day freeze dried strawberry (2.16 g/d polyphenols) with 474 mL water for 12 weeks	MDA and HNE (µmol/L)	1.8 ± 2.3	1.2 ± 0.8	NS	−0.6
Rankin, 2008 [27]	Obesity	90 g of a isocaloric placebo for 2 weeks	Urinary 8-epiPGF 2-α (pg/mg CR)	4298.2 ± 1446	3485.0 ± 1173	NS	−813.2
			ORAC_total_ (μmol/LTE)	8335.7 ± 1762	8403.1 ± 1776	0.05	67.4
		90 g of raisins for 2 weeks	Urinary 8-epiPGF 2-α (pg/mg CR)	4164.1 ± 1432.8	3107.6 ± 1069.3	<0.05	−1056.5
			ORAC_total_ (μmol/LTE)	7163.0 ± 1514.2	7614.0 ± 1609.6	<0.05	451
Ruel, 2013 [19]	Overweight	500 mL/d Placebo juice (0.08 g/d polyphenol) for 4 weeks	oxLDL (U/L)	NP	NP	NP	2.9 ± 10.9
		500 mL/d Cranberry juice (0.21 g/d polyphenol) for 4 weeks	oxLDL (U/L)	NP	NP	NP	2.3 ± 15.4

Abbreviations: NS, not significant and value not provided; NP, not provided; FRAP, ferric reducing ability of plasma; ORAC, oxygen radical absorbance capacity; TE, Trolox equivalents; MDA and HNE, malondialdehyde and hydroxynonenal; 8-epiPGF2-α, 8-epi-prostaglandin F2alpha.

**Table 6:** Effect of freeze-dried fruit polyphenols on inflammatory markers at baseline and post-intervention.

Author, Year	Risk Factors for CVD	Intervention/s	Marker Measured	Results			
				Baseline	Post-Intervention	*p*-Value	Δ
Auclair, 2010 [26]	High cholesterol	40 g/d apple powder * (0.21 g/d polyphenol) with water	CRP (mg/L)	0.98 ± 1.06	0.78 ± 0.53	NS	−0.2
		40 g/d apple powder ** (1.43 g/d polyphenol) with water	CRP (mg/L)	1.14 ± 1.08	1.16 ± 1.25	NS	+0.02
Basu, 2014 [25]	Obese and elevated serum lipids	25 g/day of a calorie/fibre matched control with 474 me water for 12 weeks	hs-CRP (mg/L)	5.1 ± 10	6.4 ± 13.9	NS	+1.3
			sVCAM-1 (ng/L)	608 ± 256	712 ± 302	NS	104
			sICAM-1 (ng/L)	226 ± 116	230 ± 116	NS	4
		25 g/day of freeze-dried strawberry (1.08 g/d of polyphenols) with water	hs-CRP (mg/L)	4.2 ± 8.5	4.4 ± 7.7	NS	0.2
			sVCAM-1 (ng/L)	709 ± 511	803 ± 410	NS	94
			sICAM-1 (ng/L)	258 ± 147	257 ± 108	NS	−1
		50 g/day of a calorie/fibre matched control with 474 mL water for 12 weeks	hs-CRP (mg/L)	4.7 ± 7.7	6.5 ± 13.9	NS	1.8
			sVCAM-1 (ng/L)	584 ± 279	776 ± 472	NS	192
			sICAM-1 (ng/L)	243 ± 124	274 ± 116	NS	31
		50 g/day of freeze dried strawberry (2.16 g/d polyphenols) with 4 water	hs-CRP (mg/L)	8.1 ± 13.9	6.9 ± 11.6	NS	−1.2
			sVCAM-1 (ng/L)	610 ± 341	768 ± 488	NS	158
			sICAM-1 (ng/L)	256 ± 178	278 ± 201	NS	22

Abbreviations: NS, not significant and value not provided; NP, not provided. (mean ± SD); * golden delicious apple powder; **, cider apple powder; CRP, C-Reactive Protein; hs-CRP, high sensitive C-Reactive Protein; sVCAM-1, soluble vascular cell adhesion molecule-1; sICAM-1, soluble intercellular adhesion molecule.

**Table 7:** Effect of other fruit polyphenols on inflammatory markers at baseline and post-intervention.

Author, Year	Risk Factors for CVD	Intervention/s	Marker Measured	Results			
				Baseline	Post-Intervention	*p*-Value	Δ
Rankin, 2008 [27]	Obesity	90 g of a isocaloric placebo for 2 weeks	CRP (mg/L)	2.20 ± 3.86	2.15 ± 3.75	NS	−0.05
			IL-6 (pg/mL)	1.23 ± 1.05	1.02 ± 0.96	NS	−0.21
			sVCAM-1 (ng/mL)	671.9 ± 151.9	678.9 ± 151.8	NS	7
			sICAM-1 (ng/mL)	209.6 ± 41.3	199.2 ± 40.4	NS	−10.4
		90 g of raisins for 2 weeks	CRP (mg/L)	2.18 ± 3.82	2.24 ± 3.94	NS	0.06
			IL-6 (pg/mL)	1.23 ± 1.05	1.08 ± 0.99	<0.05	−0.15
			sVCAM-1 (ng/mL)	657.9 ± 151.8	699.0 ± 151.9	<0.05	41.1
			sICAM-1 (ng/mL)	208.3 ± 41.3	210.0 ± 41.4	NS	1.7
Ruel, 2013 [19]	Overweight	500 mL/d of placebo juice (0.078 g/d polyphenol) for 4 weeks	sICAM-1 (ng/mL)	NP	NP	NP	14.4 ± 22.3
			sVCAM-1 (ng/mL)	NP	NP	<0.05	−14.4 ± 80.6
			sE-selectin (ng/mL)	NP	NP	NP	8.3 ± 40.2
		500 mL/d of Cranberry juice (0.2104 g/d polyphenol) for 4 weeks	sICAM-1 (ng/mL)	NP	NP	<0.05	−11.5 ± 44.6
			sVCAM-1 (ng/mL)	NP	NP	NP	−45.1 ± 119.2
			sE-selectin (ng/mL)	NP	NP	NP	−3.7 ± 7.8

Abbreviations: NS, not significant and value not provided; NP, not provided (mean ± SD); CRP, c-reactive protein; hs-CRP, high sensitive C-Reactive Protein; sVCAM-1, soluble vascular cell adhesion molecule-1; sICAM-1, soluble intercellular adhesion molecule.

**Table 8:** Effect of strawberry polyphenols on oxidative stress and inflammation six hours post-intervention.

Author, Year	Risk Factor for CVD	Intervention/s	Outcome Measure	Marker Measured	Results
					6 h Post (Least Squared Mean ± SEM)
Edirisinghe, 2011 [28]	Overweight	305 mL Strawberry beverage on one occasion with high carbohydrate moderate fat meal (94.66 ± 2.17 mg polyphenols)	Inflammation	hs-CRP (mg/L)	2.7 ± 0.1
				IL-1β (ng/L)	0.2 ± 0.0
				IL-6 (ng/L)	2.6 ± 0.2
				TNF-α (ng/L)	1.1 ± 0.1
		305 mL Placebo beverage on one occasion with high carbohydrate, moderate fat meal (2.37 ± 0.00 mg polyphenols)		hs-CRP (mg/L)	3.1 ± 0.1
				IL-1β (ng/L)	0.2 ± 0.0
				IL-6 (ng/L)	3.1 ± 0.2
				TNF-α (ng/L)	1.1 ± 0.1
Burton-Freeman, 2010 [24]	Overweight and Hyperlipidaemia	Strawberry beverage with high fat meal	Oxidative stress	OxLDL	−1.0 ± 1.5

Abbreviations: hs-CRP, high sensitive-C-Reactive Protein; IL-1β, interleukin-1beta; IL-6, interleukin-6; TNF-α, tumour necrosis factor-alpha; OxLDL, oxidative low density lipoprotein.

**Table 9:** Effect of modified grain foods on oxidative stress and inflammation post intervention.

Author, Year	Risk Factor for CVD	Intervention/s	Outcome Measure	Markers Measured	Results	
					Δ from Baseline	*p*-Value
Clerici et al., 2011 [30]	T2DM	1 serve (80 g)/d Soy germ pasta (31–33 mg isoflavones) for 8 weeks	Oxidative stress	Ox-LDL (U/mL)	−1.60 ± 2.09	NP
				8-iso-PGF2α (pg/mL)	−123.8 ± 180.4	NP
				Total antioxidant capacity (mmol/L)	79 ± 69	NP
				Glutathione (mmol/L)	+5.0 ± 2.0	NP
			Inflammation	IL-6 (pg/mL)	+0.19 ± 0.85	NP
		1 serve (80 g)/d conventional pasta	Oxidative stress	OxLDL (U/mL)	+0.83 ± 1.12	NP
				8-iso-PGF2α (pg/mL)	+71.9 ± 134.6	NP
				Total antioxidant capacity (mmol/L)	−44 ± 89	NP
				Glutathione (mmol/L)	+0.57 ± 0.93	NP
			Inflammation	IL-6 (pg/mL)	+0.98 ± 0.93	NP
Yang, 2010 [31]	Overweight/obese	4 × 40 g slices of bread/day (173 mg polyphenols)	Oxidative stress	Plasma F2-isoprostane (pmol/L)	~−20 *	NP
				Urinary F2 isoprostanes (pmol/mmol creatinine)	~+50 (450 to 500) *	NP
		4 × 40 g slices of white bread/day	Oxidative stress	Plasma F2-isoprostane (pmol/L)	~−150 *	NP
				Urinary F2 isoprostanes (pmol/mmol creatinine)	~+10 *	NP

Results provided as mean ± SD. * = Values were estimated from graphs. Abbreviations: NP, not provided; OxLDL, oxidative low density lipoprotein; 8-iso-PGF2α, 8-isoprotaglandinF2-alpha. IL-6, interleukin-6.

**Table 10:** Effect of olive oil polyphenols on oxidative stress and inflammation from baseline to post-intervention.

Author, Year	Risk Factor for CVD	Intervention/s	Outcome Measure	Marker Measured	Results	
					Δ from Baseline (Mean ± SD or Median and IQR)	*p*-Value
Ruano, 2005 [33]	High cholesterol	40 mL Olive oil (3.20 mg/day polyphenols) in a meal with 60 g white bread	Oxidative stress	8-epi-F2 α (ng/mL)	3.6 ± 0.7 to 4.5 ± 0.6	NP
		40 mL Olive oil (15.98 mg/d polyphenols) in a meal with 60 g white bread once	Oxidative stress	8-epi-F2 α (ng/mL)	4.6 ± 0.7 to 3.2 ± 0.6	NP
Widmer, 2013 [18]	Endothelial dysfunction	30 mL uncooked olive oil/d with one of their usual meals for 4 months (10.2 mg/d polyphenols)	Oxidative stress	OxLDL (mg/dL)	4.29 ± 2.86 to 4.31 ± 2.77	0.79
				Plasma 8-isoprostanes	130.8 (28.71, 310.6) to 164.4 (55.37, 491.6)	0.003
			Inflammation	ICAM-1 (pg/mL)	196 (84, 298) to 183 (91, 239)	<0.001
				VCAM-1 (pg/mL)	575 (368, 1054) to 560 (346, 1166)	0.068
				IL-6 (pg/mL)	1.3 (0.3, 33) to 1.3 (0.5, 5.1)	0.518
				hs-CRP (mg/L)	0.101 (0.01, 2.81) to 0.09 (0.01, 1.21)	0.220
Moreno-Luna, 2012 [32]	High-normal BP or stage 1 essential hypertension	60 mL (30 mg/d) polyphenol rich olive oil with Mediterranean style diet for 2 months	Oxidative stress	Ox-LDL (µg/L)	−28.2 ± 28.5	NP
			Inflammation	hs-CRP (mg/L)	−1.9 ± 1.3	NP
		60 mL polyphenol free olive oil with Mediterranean style diet for 2 months	Oxidative stress	Ox-LDL (µg/L)	−6.9 ± 22.2	NP
			Inflammation	hs-CRP (mg/L)	−0.6 ± 0.9	NP

Results presented as Mean ± SD or median (interquartile range). Abbreviations: NP, not provided; OxLDL, oxidative low density lipoprotein; ICAM-1, intercellular adhesion molecule-1; VCAM-1, vascular cell adhesion molecule-1. IL-6, interleukin-6; hsCRP, high sensitive C-Reactive Protein.

**Table 11:** Effect of tea polyphenols on oxidative stress and inflammation.

Author, Year	Risk Factor for CVD	Intervention/s	Outcome Measure	Marker Measured	Results	
					Δ from Baseline *	*p*-Value
De Maat, 2000 [34]	Smoking	Black tea for 4 weeks	Inflammation	CRP (mg/L)	−0.6 (2.5)	NP
				IL-6 (pg/mL)	+2.48(7.32)	NP
				IL-1β (pg/mL)	+0.42 (1.65)	NP
				TNF-α (pg/mL)	+0.10 (0.81)	NP
		Green tea for 4 weeks	Inflammation	CRP (mg/L)	+0.9 (1.8)	NP
				IL-6 (ng/L)	+0.64 (1.40)	NP
				IL-1β (ng/L)	−0.87 (2.05)	NP
				TNF-α (ng/L)	−0.23 (0.84)	NP
		Green tea isolate for 4 weeks	Inflammation	CRP (mg/L)	−0.2 (2.1)	NP
				IL-6 (ng/L)	−0.11 (0.46)	NP
				IL-1β (ng/L)	−0.04 (0.29)	NP
				TNF-α (ng/L)	−0.24 (0.96)	NP
		Water for 4 weeks	Inflammation	CRP (mg/L)	−0.6 (2.2)	NP
				IL-6 (ng/L)	+0.28 (2.67)-	NP
				IL-1β (ng/L)	+0.06 (0.42)	NP
				TNF-α (ng/L)	−0.09 (0.69)	NP
Hakim, 2004 [35]	Smoking	Decaffeinated green tea for 4 months	Oxidative stress	Urinary 8-OHdG (ng/mg creatinine)	−1.6 ± 2.0	0.44
		Decaffeinated black tea for 4 months	Oxidative stress	Urinary 8OHdG (ng/mg creatinine)	2.7 ± 2.2	0.23
		Water for 4 months	Oxidative stress	Urinary 8-OHdG (ng/mg creatinine)	2.64 ± 1.9	0.20

* Δ from baseline data expressed as mean ± SEM or mean (SD) or for logarithmically transformed data geometric mean (CV). Abbreviations: CRP, C-Reactive Protein; IL-6, interleukin-6; IL-1β, interleukin-1beta; TNF-α, tumour-necrosis factor-alpha.

**Table 12:** Effect of wine on oxidative stress.

Author, Year	Risk Factor for CVD	Intervention/s	Marker Measured	Results	
				Δ from Baseline (Mean ± SEM)	*p*-Value
Abu-Amsha Cacetta, 2001 [36]	Smoking	Dealcoholised red wine for 2 weeks (454.5 mg/d)	Plasma F2-sioprostane (pmol/L)	882.5 ± 59.6 to 703.4 ± 49.2	NS
			Urinary F2-isoprostane (pmol/mmol creatinine)	272.6 ± 30.9 to 255.7 ± 19.8	NS
		375 mL Red wine for 2 weeks (450 mg polyphenols/d)	Plasma F2-sioprostane (pmol/L)	810.4 ± 57.1 to 811.3 ± 43.0	NS
			Urinary F2-isoprostane (pmol/mmol creatinine)	275.0 ± 22.8 to 296.2 ± 29.0	NS
		375 mL (129 mg polyphenols/d) White wine for 2 weeks	Plasma F2-sioprostane (pmol/L)	867.5 ± 54.8 to 831.7 ± 79.0	NS
			Urinary F2-isoprostane (pmol/mmol creatinine)	271.1 ± 21.9 to 286.3 ± 25.2	NS

NS, not significant and values not provided. Abbreviations: SEM, standard error of the mean; CVD, cardiovascular disease.

## Supplementary Materials

The following are available online at www.mdpi.com/2227-9032/4/3/69/s1, to support the review methodology.
