# Supplementary material for: Effect of Flavonoids on Oxidative Stress and Inflammation in Adults at Risk of Cardiovascular Disease: A Systematic Review
Source: Healthcare (Basel). 2016 Sep 14;4(3):69. doi: 10.3390/healthcare4030069 (PMC5041070; doi:10.3390/healthcare4030069)
Supplement: Supplementary file 1 [file healthcare-04-00069-s001.pdf]

Supplementary Materials: Effect of Flavonoids on Oxidative Stress and Inflammation in Adults at Risk of Cardiovascular Disease: A Systematic Review

Jenni Suen, Jolene Thomas, Amelia Kranz, Simon Vun and Michelle Miller

In reference to Section 2.2 Search Strategy (page 3 of the manuscript), the following search strategies were applied to each database:

Medline

#	Searches	Results
1	* Polyphenols	1238
2	Limit 1 to (English language and humans)	594
3	Polyphenol.mp. (mp = title, abstract, original title, name of substance word, subject heading word, keyword heading word, protocol supplementary concept word, rare disease supplementary concept word, unique identifier)	6327
4	Limit 3 to (English language and humans)	2215
5	Flavonoid.mp (mp = title, abstract, original title, name of substance word, subject heading word, keyword heading word, protocol supplementary concept word, rare disease supplementary concept word, unique identifier)	10,924
6	Limit 5 to (English language and humans)	3212
7	* flavonoids/ or * anthocyanins/ or * benzoflavones/ or * bioflavonoids/ or * catechin/ or * chalcones/ or * flavanones/ or * flavones/ or * flavonolignans/ or * flavonols/ or * isoflavones/ or * proanthocyanidins/	35,617
8	Limit 7 to (English language and humans)	12,245
9	* Oxidative Stress/	37,608
10	Limit 9 to (English language and humans)	17,936
11	* Inflammation/	40,537
12	Limit 11 to (English language and humans)	23,251
13	Oxidative stress.mp (mp = title, abstract, original title, name of substance word, subject heading word, keyword heading word, protocol supplementary concept word, rare disease supplementary concept word, unique identifier)	127,345
14	Limit 13 to (English language and humans)	56,835
15	Inflammat *.mp (mp = title, abstract, original title, name of substance word, subject heading word, keyword heading word, protocol supplementary concept word, rare disease supplementary concept word, unique identifier)	678,948
16	Limit 14 to (English language and humans)	393,710
17	* Adult/	523
18	Limit 16 to (English language and humans)	265
19	Adult.mp (mp = title, abstract, original title, name of substance word, subject heading word, keyword heading word, protocol supplementary concept word, rare disease supplementary concept word, unique identifier)	4,443,010
20	Limit 18 to (English language and humans)	3,351,279
21	2 or 4 or 6 or 8	15,243
22	10 or 12 or 14 or 16	438,394
23	18 or 20	3,351,279
24	21 and 22	2757
25	23 and 24	308

* Prior to the search term means that the term was searched as a MeSH term; * Post term is a truncation of the term to enable multiple endings of the term to be included. E.g. inflammtat * includes inflammation, inflammatory etc.

Cochrane Library

**Title, Abstract, Keywords**		(Polyphenol or Flavonoid or Anthocyanin or Catechin or Flavon* or isoflavon* or benzoflavone or proanthrocyanidin)
**And**	**Title, Abstract, Keywords**	Oxidative stress or inflammat*
**And**	**Search All Text**	Adult or Aged

All Results: 289
Cochrane Reviews: 2
Trials: 285

Cinahl

#	**Search**	**Search Options**	**Results**
1	TX polyphenol or TX flavonol or TX anthrocyanin or TX isoflavn* or TX benzoflavone or TX proanthrocyandin	Search modes-Boolean/Phrase	2055
2	TX oxidative stress or TX inflammat*	Search modes-Boolean/Phrase	46,185
3	1 and 2	Search modes-Boolean/Phrase	304
4	TX adult	Search modes-Boolean/Phrase	752,195
5	TX adult or TX aged	Search modes-Boolean/Phrase	771,922
6	3 and 5	Search modes-Boolean/Phrase	78

Scopus

**(Polyphenol or Flavonoid or Anthocyanin or Catechin or Flavon* or isoflavon* or benzoflavone or proanthrocyanidin)**		**Article Title, Abstract, Keywords**
And	Oxidative stress or inflammat*	Article Title, Abstract, Keywords
And	Adult or Aged	Article Title, Abstract, Keywords

Limit to: language, "English" and exactkeyword, "Human"
Results: 573

All articles found from the database searches above were imported into an Endnote database. The articles were then filtered as per Figure 2 on page 5 of the manuscript.

In reference to 2.5 Quality assessments, the final Cochrane Collaboration quality assessment tables below were used report on the quality of the studies. The tables below include collated points and judgment of both reviewers.

**The Cochrane Collaboration's Tool for Assessing Risk of Bias**			**Study Design: Double-Blinded Randomised Controlled Cross over**
**Study Details:**<br>Mellor, D.D.; Madden, L.A.; Smith, K.A.; Kilpatrick, E.S.; Atkin, S.L. High-polyphenol chocolate reduces endothelial dysfunction and oxidative stress during acute transient hyperglycaemia in Type 2 diabetes: A pilot randomized controlled trial. *Diabet. Med.* **2013**, *30*, 478–483.			
**Domain**	**Support for Judgment**		**Review Authors' Judgment**
Selection bias			
**Random sequence generation**	States randomization code was held at chocolate provider (Barry Callebaut) (p. 480).		Unclear risk of bias as method of generating the randomization code was not provided. Therefore not enough information is provided to determine if method used is at risk of bias.
**Allocation concealment**	"Barry Callebaut provided both chocolates in identical presentation". (p. 480).		This suggests that allocation concealment may have occurred, however no information was provided on allocation concealment. Unclear risk of bias.
Performance bias			
**Blinding of participants and personnel**	States it is a double-blinded study.		Low risk of bias.
(Assessments should be made for each main outcome or class of outcomes)	Intervention was identical in appearance, composition (exception of polyphenol content) and packaging. Only potential is a difference in taste which was not mentioned, thus likelihood is low.		
Detection bias			
**Blinding outcome assessment**	States it is a double-blinded study.		Low risk of bias.
(Assessments should be made for each main outcome or class of outcomes)	Says that they are blinded but due to lack of information, unsure if method used disables researchers awareness of the intervention provided. However if not blinded properly, unlikely to affect results, as they are objective measures.		
Attrition bias			
**Incomplete outcome data**	No dropouts reported. Data reported for the 10 participants that underwent the randomization and allocation as evidenced by flow chart on page 479.		Low risk of bias, as data was reported for all 10 participants.
(Assessments should be made for each main outcome or class of outcomes)	Excluded one participant at screening due to anaemia (p. 479).		
Reporting bias			
**Selective reporting**	**Outcomes as per methods**	**Reported in results (Yes/No)**	Low risk of bias as all outcomes reported as per the study method.
	Endothelial function measured by the EndoPAT.	Yes (p. 480).	

	Oxidative stress measured by Urinary 25-F2t isoprostane: creatinine.	Yes (p. 480).	
Other bias			
**Other sources of bias**	Carry over effect "One month prior to crossover" (p. 479).		Low risk of bias due to the following:<br>- Considered sources of confounding such as diet and physical activity which ensures participants have the same diet before each intervention period and assessed adherence to this diet.<br>- Considered potential carry over effect and implemented a washout period.<br>- Suggest that industrial funding does not influence results.<br>- Risk of type 1 error is 0.03 as changes in endothelial function observed are smaller than anticipated (0.6 seen vs. 1.8 anticipated) however confidence intervals are quite small suggesting that effect is present but may not be statistically significant.
	Confounding: "2-week pre-start washout period where they abstained from rich sources of polyphenol (using a list of foods provided) and omitted all chocolate and cocoa" (p. 479). 1 week post intervention period 1 washout (p. 480). States: "To assess dietary adherence and reduce the potential confounding resulting from a change in background diet, dietary intake was recorded using 24-h dietary recall by study dietitian. (p. 480)." State: "Dietary analysis and assessment of physical activity levels showed no significance intra-subject differences between the two groups." (p. 481).		
	Power calculation: "A power calculation was undertaken based upon the data of Balzer et al using G* Power which suggested a minimum sample size of seven (based on a difference of 1.8 in endothelial function, power = 0.80 for alpha <0.05). Fasting endothelial function was 1.7 ± 0.1 and 2.3 ± 0.1 180 min after chocolate consumption. With a % change p = 0.03)."		
	Source of funding: "funded by Barry Callebaut Beglium NV, but study design and analysis were undertaken independently by the research team." Did not declare and competing interests. (p. 482).		
	Site of recruitment: Not stated.		
	Adherence or compliance: "To assess dietary adherence and reduce the potential confounding resulting from a change in background diet" (p. 480).		
**Overall risk of bias**			Low risk of bias considering that all data collected was objective measures that were all reported. Study design controlled for potential confounder and carry over effect. Study design suggests adequate participant blinding as chocolate was provided by chocolate provider in identical presentation. Slight risk of potential selection and detection bias may have occurred due to the inadequate information provided but risk considered small as all outcomes were reported.

**The Cochrane Collaboration's Tool for Assessing Risk of Bias**		**Study Design: Randomised Single-Blinded Cross over Study**
**Study Details:** Carnevale, R.; Loffredo, L.; Pignatelli, P.; Nocella, C.; Bartimoccia, S.; di Santo, S.; Martino, F.; Catasca, E.; Perri, L.; Violi, F. Dark chocolate inhibits platelet isoprostanes via NOX2 down-regulation in smokers. *J. Thromb. Haemost.* **2012**, *10*, 125–132.		
**Domain**	**Support for judgment**	**Review authors' judgment**
Selection bias		
**Random sequence generation**	"They were randomly allocated to a treatment sequence with 40 g of dark chocolate (≥85% cocoa) or milk chocolate (≤35% cocoa in a cross over, single blind design' (p. 126).<br>'The randomization was carried out by a procedure based on a random numeric sequence" (p. 126).	Low risk of bias as random numeric sequence was used.
**Allocation concealment**	"An individual not involved in the study, assigned codes to the study treatments, randomly allocated the participants to a treatment sequence with dark or milk chocolate and kept the key in sealed envelope."<br>"The authors and laboratory technicians were unaware of the treatment allocation." (p. 126).	Low risk of bias as individual not involved in the study conducted allocation and used sealed, key kept in envelope and states that investigators measuring outcome were unaware of allocation.
Performance bias		
**Blinding of participants and personnel**<br>(Assessments should be made for each main outcome or class of outcomes)	"Intrinsic difficulties in performing a double-blind study with dark and milk chocolate. (p. 131)."	High risk of bias as method to mask the different appears of treatment was not conducted.
Detection bias		
**Blinding outcome assessment**<br><br>(Assessments should be made for each main outcome or class of outcomes)	States: "blind laboratory analysis" (p. 131) and "single blinded study" (p. 126).	Low risk of bias as this suggests that laboratory technicians who have not collected the data, conducted the laboratory analysis. This reduces any potential risk of bias associated with unmasked participants accidently expressing their treatment allocation to investigators.
Attrition bias		
**Incomplete outcome data**	Dropouts not mentioned in methods and result	Unclear risk of bias as no dropouts and number of

(Assessments should be made for each main outcome or class of outcomes)	(pp. 126–127).<br>Number of participants in analysis not stated in results (pp. 127–129)		participants used in analysis not stated (p. 128).
Reporting bias			
**Selective reporting**	**Outcomes as per methods**	**Reported in results (Yes/No)**	Reported on all outcome measures reported.<br>Low risk of bias
	Platelet function	Yes (p. 128)	
	Oxidative stress measured by Platelet 8-iso-PGF2α assay	Yes (p. 128)	
Other bias			
**Other sources of bias**	Carry over effect: "There was an interval of 7 days between the two phases of the study." (p. 125).		Suggest low risk of bias as study:<br>- Reduced carry over effect by providing a washout period between interventions<br>- Considered impact of calories from different chocolates being a source of confounding<br>- SD observed in outcome used for power calculation met prediction and this probability of type 1 error is 0.05<br>- Source of funding was not stated but authors declare no conflict of interest.<br>- Measuring adherence to study product was not applicable as intervention only provided on one occasion and provided by investigators.<br>No dietary assessment to measure compliance polyphenol-free diet in 24 h prior to measurement.
	Confounding: "Furthermore, there were no significant differences in caloric content between the dark (Calories 230) and milk (Calories 220) chocolate." (p. 126).		
	Power calculation: "...difference in platelet sNOX2-dp variation in smokers to be detected between dark and milk chocolate treatments and paired differed SD = 5 and type I error probability =0.05 and power 1 − β = 0.90. n = 12" (p. 127).		
	Source of funding: "The authors state that they have no conflict of interest" (p. 131).		
	Site of recruitment: Not stated.		
	Adherence/compliance: Not stated		
**Overall risk of bias**			Low risk of bias as unmasked participants are unlikely to affect objective outcome measures assessed. As dropouts were not reported, it's likely there were no dropouts and analysis was performed on all participants.

**The Cochrane Collaboration's Tool for Assessing Risk of Bias**		**Study Design: Randomised, Placebo-Controlled Double-Blind Cross over Study**
**Study Details:** Mellor, D.D.; et al. High-cocoa polyphenol-rich chocolate improves HDL cholesterol in Type 2 diabetes patients. *Diabet. Med.* **2010**, *27*, 1318–1321.		
**Domain**	**Support for judgment**	**Review authors' judgment**
Selection bias		
**Random sequence generation**	'Randomisation was undertaken by Nestec Ltd with enough chocolate being given to subjects for 8-week period (p. 1319)'.	Unclear risk of bias as method of randomisation is not provided.
**Allocation concealment**	Not stated	Unclear risk of bias as information of allocation concealment was not stated.
Performance bias		
**Blinding of participants and personnel** (Assessments should be made for each main outcome or class of outcomes)	States it's a double-blinded study (p. 1318), "dyed to the same colour as high polyphenol chocolate" (p. 1319). "a blinded taste study was undertaken prior to the trial that showed that the subjects could not tell any difference in appearance or taste between the high-polyphenol chocolate and the low-polyphenol chocolate preparations (p. 1320)."	Low risk of bias as blinded taste test was conducted.
Detection bias		
**Blinding outcome assessment** (Assessments should be made for each main outcome or class of outcomes)	States it's a double-blinded study (p. 1318).<br>Objective outcome assessment performed:<br>- fasting blood samples of total cholesterol, triglyceride and HDL cholesterol levels, plasma glucose, serum insulin, HbA1c, CRP<br>- Blood pressure<br>- Weight	Although method of blinding is not reported, due to the objective nature of the outcome measures assessment, detection is unlikely to affect the results.<br>Low risk of bias
Attrition bias		

**Incomplete outcome data**<br>(Assessments should be made for each main outcome or class of outcomes)	Drop outs not mentioned in methods or results (pp. 1318–1319). "twelve subjects were enrolled and completed the study" (p. 1318). All twelve study participants completed the trial with no drop-outs and no reported missing data for either objective or subjective outcomes. The number of participants randomised to the treatment and control groups is not reported, however this is unlikely to influence the results due to the crossover design.	Low risk of bias
Reporting bias		
**Selective reporting**	**Outcomes as per methods** / **Reported in results (Yes/No)**<br>Weight / Yes (p. 1320)<br>Glycaemic control / Yes (p. 1320)<br>Lipid profile / Yes (p. 1320)<br>High-sensitivity CRP / Yes (p. 1320)	All outcomes intended in methods were reported.<br>Low risk of bias<br>3-month lipids checked, no difference and not reported.
Other bias		
**Other sources of bias**	Carry over effect: States: "crossed over after 4week washout period (p. 1318)."<br>Confounding: States: "Subjects were advised not to consume any other chocolate for the duration of the study, apart from this, subject were instructed to make no further changes to their diet and lifestyle (p. 1318)."<br>Power calculation: "At $p < 0.05$ level of significance, a sample size of 12 subjects in a crossover fashion will provide >90% power to detect a 0.4 mmol/L change in plasma HDL cholesterol concentration." (p. 1319).<br>Source of funding: "The chocolate for the study was provided as an unrestricted gift from Nestle PTC, York and was funded through the Diabetes Research and Development fund (p. 1318)."<br>Site of recruitment: Not stated.<br>Adherence/compliance: "To monitor compliance, subjects were asked to return all empty wrappers, noting the time and date when it was consumed on the wrapper. (p. 1319)."	Low risk of bias as study accounted for:<br>- Carry over effects<br>- Confounding due to diet and lifestyle<br>- Change of >0.4mmol/L seen in plasma HDL.<br>- Assess adherence<br>- Intervention product provided as a gift
**Overall risk of bias**		Low risk of bias

The Cochrane Collaboration's Tool for Assessing Risk of Bias		Study Design: Randomised, Controlled, Cross-over, Free-Living Study
**Study Details:** Sarria, B.; Martinez-Lopez, S.; Sierra-Cinos, J.L.; Garcia-Diz, L.; Mateos, R.; Bravo, L. Regular consumption of a cocoa product improves the cardiometabolic profile in healthy and moderately hypercholesterolaemic adults. *Br. J. Nutr.* **2014**, *111*, 122–134.		
**Domain**	**Support for judgment**	**Review authors' judgment**
Selection bias		
**Random sequence generation**	"Randomised, controlled, cross-over, free-living study (p. 122)".	Unclear risk of bias as method of randomization is not provided.
**Allocation concealment**	Not stated	Unclear risk of bias as information not provided
Performance bias		
**Blinding of participants and personnel** (Assessments should be made for each main outcome or class of outcomes)	"The lack of blinding of subjects and investigators may have led to certain bias" (p. 132). Participants may have altered their diet depending on treatment, however background diet was controlled for as a confounder and the crossover design would minimise the effect of this on the results.	Unclear risk of bias
Detection bias		
**Blinding outcome assessment** (Assessments should be made for each main outcome or class of outcomes)	"the lack of blinding of subjects and investigators may have led to certain bias" (p. 132) However this is unlikely to influence the results as all outcome measures are objective.	Low risk of bias
Attrition bias		
**Incomplete outcome data** (Assessments should be made for each main outcome or class of outcomes)	"…six withdraw due to personal, health or professional reasons (p. 126). Results as per tables provided results for only the 44 participants that completed the study (pp. 129–130)."	Low risk of bias as intention to treat analysis not required for due to cross over design and results table suggests that all participants that completed the study were included in the analysis.
Reporting bias		
**Selective reporting**	**Outcomes as per methods** / **Reported in results (Yes/No)**<br>Serum lipid lipoprotein profile / Yes (p. 128)<br>Oxidative stress / Yes (p. 130)	Low risk of bias as reported on all outcomes measured

	Inflammatory markers Yes (p. 129)	
	Blood pressure Yes (p. 130)	
Other bias		
**Other sources of bias**	Carry over effect: Wash out period not stated	Unclear risk of bias due to potential carry over effect
	Confounding: "After a 2-week run-in stage, in which consumption of the fruit, vegetables and beverage mentioned below was restricted." (p. 124). Their dietary intake was regularly evaluated to control any possible changes. (p. 124).	
	Power calculation: Not stated	
	Source of funding: Not stated	
	Site of recruitment: 'Volunteer recruitment was carried out by placing advertisements in the Universidad Complutense campus as well as by giving short talks between lectures.' (p. 123).	
	Adherence/compliance: "Compliance was controlled by counting the number of cocoa servings provided to the volunteers before and after the interventions, as well as by weekly calling the volunteers. (p. 124)."	
**Overall risk of bias**		Unclear risk of bias due to source of performance and detection bias and potential carry over effects

**The Cochrane Collaboration's Tool for Assessing Risk of Bias**		**Study Design: Randomised Double-Blinded Cross over Trial**
**Study Details:** Ruel, G.; Lapointe, A.; Pomerleau, S.; Couture, P.; Lemieux, S.; Lamarche, B.; Couillard, C. Evidence that cranberry juice may improve augmentation index in overweight men. *Nutr. Res.* **2013**, *33*, 41–49.		
**Domain**	**Support for judgment**	**Review authors' judgment**
Selection bias		
**Random sequence generation**	States that "randomly assigned to drink 500 mL CJC/day (27% juice) or 500 mL placebo juice (PJ)/day for 4 weeks..." (p. 41)	Unclear risk of bias as method of randomization not provided.
**Allocation concealment**		Unclear risk of bias as information on potential allocation concealment not provided.
Performance bias		
**Blinding of participants and personnel** (Assessments should be made for each main outcome or class of outcomes)	States that it is a double blind study (p. 41). "The CJC and PJ used in the present study had similar organoleptic properties (taste, colour and texture) and vitamin C contents but no cranberries entered in the parathion of the PJ. (p. 42)". "Both juices were packaged at Universite Laval in 125 mL ready-to drink TetraBrik boxes under the close monitoring of Ocean Spray to ensure adequate reconstitution and quality of the juices (p. 42)".	Low risk of bias as both interventions was similar in appearance.
Detection bias		
**Blinding outcome assessment** (Assessments should be made for each main outcome or class of outcomes)	States that it is a double blind study (p. 41).	Information on how investigators were masked was not provided but the results are unlikely to be affected if blinding was broken as the outcome measures are objective. Low risk of bias.
Attrition bias		
**Incomplete outcome data** (Assessments should be made for each main outcome or class of outcomes)	No dropouts reported. As per the result tables on pages 43–47, all participants were accounted for. No intention to treat analysis.	Low risk of bias as all outcome data collected was presented.
Reporting bias		

**Selective reporting**	Outcome measured is AIx and cardiometabolic profile (p. 42). Not stated in methods what parameters are measured for the cardiometabolic profile (pp. 42–43).	Unclear risk of bias.
Other bias		
**Other sources of bias**	Carry over effect: "Upon entry into the study, subjects were instructed by a dietician to maintain their usual nutritional habits, limit their alcohol consumption to a maximum of 1 drink per day as well as restrain themselves from consuming any vitamin, antioxidant or mineral supplements. (p. 42)" "following a run-in period of 4 weeks during which participants were asked to drink 500ml of water a day in order to get the subjects acquainted with the introduction of such an amount of liquid into their usual diet." (p. 42). "After a 4 week washout period (500 mL water/d), treatments were crossed over." (p. 42).	Unclear risk of bias as study design aimed to reduce carry over effects but not effects from potential confounding and adherence to intervention products.
	Confounding.	
	Power calculation: Not stated.	
	Source of funding: Canadian Institutes of Health Research. It is made clear that the organisations providing funding were not involved in the design or conduct of the study.	
	Site of recruitment: "through media" (p. 42).	
	Adherence/compliance: Not stated.	
**Overall risk of bias**		Unclear risk of bias of selection, detection, reporting and other bias due to lack of information provided.

**The Cochrane Collaboration's Tool for Assessing Risk of Bias**		**Study Design: Randomised Dose-Response Controlled Trial**
**Study Details:** Basu, A.; Betts, N.M.; Nguyen, A.; Newman, E.D.; Fu, D.; Lyons, T.J. Freeze-dried strawberries lower serum cholesterol and lipid peroxidation in adults with abdominal adiposity and elevated serum lipids. *J. Nutr.* **2014**, *144*, 830–837.		
**Domain**	**Support for judgment**	**Review authors' judgment**
Selection bias		
**Random sequence generation**	"Randomly assigned to consume 1 of the following 4 beverages for 12 week" (p. 831).	Unclear risk of bias due to lack of information provided on randomization method.
**Allocation concealment**	Not stated	Unclear risk of bias due to lack of information provided
Performance bias		
**Blinding of participants and personnel** (Assessments should be made for each main outcome or class of outcomes)	"In addition, the control beverages contained added red food colour (McCormick & Company) and artificial strawberry-flavoured Kool-Aid (Kraft Foods) to mimic the colour and flavor of the FDS beverages. (p. 831). Absence of placebo agent that is identical to the FDS powder and could be used in a double-blind treatment (p. 835)."	Unclear risk of bias as this suggests that strategies to mask participants were put in place but detectable authors suggest that there may be detectable differences.
Detection bias		
**Blinding outcome assessment** (Assessments should be made for each main outcome or class of outcomes)	"All laboratory staff were unaware of the treatment groups. (p. 832)."	Low risk of bias
Attrition bias		
**Incomplete outcome data** (Assessments should be made for each main outcome or class of outcomes)	Not stated if intention to treat analysis was performed in methods or results (pp. 831–833). 85 participants tested, 66 met inclusion criteria (6 dropped out due to time constraints and 60 completed the study protocol. Not stated how many participants were initially randomised however reasons for drop outs are unrelated to the outcomes of interest and unlikely to affect the results. Data from all 60 participants appear to have been reported with no missing data.	Low risk of bias
Reporting bias		
**Selective reporting**	Reported on all outcomes anticipated.	Low risk of bias

Other bias		
**Other sources of bias**	Carry over effect: not stated: N/A due to parallel design.	Low risk of bias due to study design accounting for<br>- Sources of confounding<br>- Adherence to intervention<br>- Potential carry over effects not applicable due to parallel design<br>- Sample size meet and changes in TC and LDL observed
	Confounding: "The participants were instructed to add the strawberry or control beverages as a snack to their usual diet and not to replace it with any meals." "Asked to refrain from consuming any other source of berries or related products derived from berries, such as juices, jams and desserts. Also asked to refrain from consuming green tea, cocoa and soy products while participating in the study. (pp. 831–832)." "Participants were instructed to maintain their usual diet, physical activity, and lifestyle while in the study. (p. 832)." "Control beverages were matched for calories and total fibre (p. 830)."	
	Power calculation: "Target sample size was calculated to include 15 participants per group to detect minimum differences of 0.3 mmol/L in serum total cholesterol and 0.2 mmol/L in LDL cholesterol with 80% power based on out previous feasibility study" (p. 3).	
	Source of funding/ conflict of interest: "received monetary compensation during these weekly visits. (p. 831)."	
	Site of recruitment: "Clinical Research Center in University of Oklahoma Health Science Centre and Nutritional Sciences Clinical Assessment Unit at Oklahoma State University. (p. 831)."	
	Adherence/compliance: "required to make 3 visit/wk to their study site to ensure compliance by supervised consumption on these days (p. 831)." "return any unconsumed strawberry and control beverages (p. 831)."	
**Overall risk of bias**		Low risk of bias

**The Cochrane Collaboration's Tool for Assessing Risk of Bias**			**Study Design: Randomised, Single-Blinded, Placebo Controlled, 12 Week Cross over Trial**
**Study Details:** Burton-Freeman, B.; Linares, A.; Hyson, D.; Kappagoda, T. Strawberry modulates LDL oxidation and postprandial lipemia in response to high-fat meal in overweight hyperlipidemic men and women. *J. Am. Coll. Nutr.* **2010**, *29*, 46–54.			
**Domain**	**Support for judgment**		**Review authors' judgment**
Selection bias			
**Random sequence generation**	"Randomised single-blind, placebo-controlled, 12 week crossover trial (p. 46)"		Unclear risk of bias as method of randomization not reported
**Allocation concealment**	Not stated		Unclear risk of bias as allocation concealment not reported
Performance bias			
**Blinding of participants and personnel** (Assessments should be made for each main outcome or class of outcomes)	States its single-blind (p. 46)		Suggests that participants are masked but method not reported. Unclear risk of bias
Detection bias			
**Blinding outcome assessment** (Assessments should be made for each main outcome or class of outcomes)	Not stated		Suggests that investigators we not masked but all outcome assessments were objective and thus lack of blinding should theoretically have little effect on the results.<br>Unclear risk of bias
Attrition bias			
**Incomplete outcome data** (Assessments should be made for each main outcome or class of outcomes)	Table on page 51 states "*n* = 24". "Twenty-four hyperlipidaemic men and women were recruited… (p. 46)". There were 2 dropouts due to work commitments and caffeine withdrawal on postprandial testing days and there data was not included in the analysis.		Low risk of bias as all participants finished the trial and was included in the analysis. Dropouts were unrelated to study intervention. The inclusion of drop out data would have diluted the results.
Reporting bias			
**Selective reporting**	**Outcomes as per methods**	**Reported in results (Yes/No)**	Low risk of bias
	Oxidative stress	Yes (pp. 50–51)	
Other bias			

**Other sources of bias**	Carry over effect: "Subjects were transitioned immediately from one beverage to the next based on sequence randomization with no formal washout at crossover (p. 47)."<br>Confounding: "10-day run-in period (p. 46)." "… to establish that there were no unanticipated changes in subjects' diets during the study period. (p. 48)." The background diet of the subject was berry free for the duration of the intervention, but was not otherwise controlled for other food high in antioxidants and polyphenols. Vitamin C content was lower on the Pbo treatment compared to intervention treatment.<br>Power calculation: Not stated.<br>Source of funding: Funded by the California Strawberry Commission.<br>Site of recruitment: Sacremento, California, community and surrounding region were recruited using newspaper and online advertisements and local flyers (p. 47).<br>Adherence/compliance: "During the two 6-week feeding periods, subjects returned to the testing center at biweekly intervals to pick up the Str or Pbo beverages and for a brief assessment of study adherence (p. 48)."	Method of measuring adherence and addressing in changes in diet reduces the risk of bias. However without a washout period, not controlling for physical activity lower vitamin c (antioxidant) content in placebo and funding from industry, this study puts this study at high risk of bias.
**Overall risk of bias**		Unclear risk of bias secondary to unmasked investigators and no method to reduce potential carry over effects.

**The Cochrane Collaboration's Tool for Assessing Risk of Bias**		**Study Design: Single-Centre, Randomised, Single Blinded, Placebo-Controlled Cross-over Trial**
**Study Details:** Edirisinghe, I.; Banaszewski, K.; Cappozzo, J.; Sandhya, K.; Ellis, C.L.; Tadapaneni, R.; Kappagoda, C.T.; Burton-Freeman, B.M. Strawberry anthocyanin and its association with postprandial inflammation and insulin. *Br. J. Nutr.* **2011**, *106*, 913–922.		
**Domain**	**Support for judgment**	**Review authors' judgment**
Selection bias		
**Random sequence generation**	"During the experiment, the subjects consumed two test meals in random order, with each subject serving as his/her own control. (p. 914)".	Unclear risk of bias as method of randomization not reported.
**Allocation concealment**	Not stated.	Unclear risk of bias as allocation concealment not reported
Performance bias		
**Blinding of participants and personnel** (Assessments should be made for each main outcome or class of outcomes)	States it was single-blinded.	Suggests that participants were masked but method of masking was not reported Treatment both matched of volume, favour, and nut contribution. Low risk of bias
Detection bias		
**Blinding outcome assessment** (Assessments should be made for each main outcome or class of outcomes)	Not stated.	Investigators not blinded but outcomes are objective measures and the cross over design reduces the risk of detection affecting the results. Low risk of bias
Attrition bias		
**Incomplete outcome data** (Assessments should be made for each main outcome or class of outcomes)	"Of the sixteen women, two dropped out of the study because of work commitments. (p. 914)".	No intention to treat analysis and not stated at which stage did the participants drop out but cross over design so Low risk of bias
Reporting bias		
**Selective reporting**	Baseline inflammatory markers not reported (p. 919). Only looked at between group differences.	Unclear risk of bias<br>Low risk of bias
Other bias		

**Other sources of bias**	Carry over effect: "Briefly, the subject reported to the laboratory in the morning in a fasting state on two occasions 3–5 days apart (p. 914)."<br>Confounding: "Eligible subjects had a 7 day run-in before the actual experiment during which they were required to avoid consuming berries, including strawberries, while mainting all other aspects of their diet and physical activity. (p. 914)."<br>Power calculation: Not stated.<br>Source of funding: Funded by strawberry commission.<br>Site of recruitment: Sacramento, CA, USA community.<br>Adherence/compliance: N/A as on one occasion	Unclear risk of bias
**Overall risk of bias**		Unclear risk of bias

**The Cochrane Collaboration's Tool for assessing risk of bias**			**Study Design: Randomised, Cross over Design**
**Study Details:** Rankin, J. W.; Andreae, M.C.; Chen, C.Y.O.; O'Keefe, S.F. Effect of raisin consumption on oxidative stress and inflammation in obesity. *Diabetes Obes. Metab.* **2008**, *10*, 1086–1096.			
**Domain**	**Support for judgment**		**Review authors' judgment**
Selection bias			
**Random sequence generation**	States: "A randomised, counterbalanced, cross design was used in order to have subjects undergo raisin and isoenergic placebo treatments (p. 1087)."		Unclear risk of bias as method of randomization not reported
**Allocation concealment**	Not stated		Unclear risk of bias as allocation concealment not reported
Performance bias			
**Blinding of participants and personnel** (Assessments should be made for each main outcome or class of outcomes)	Not stated		Blinding not used due to the nature of the intervention, intervention = raisins, placebo = jelly candies. All participants were exposed to both treatments due to cross over design and therefore it is unlikely that lack of blinding would have influenced the results but Unclear risk of bias
Detection bias			
**Blinding outcome assessment** (Assessments should be made for each main outcome or class of outcomes)	Not stated		Lack of blinding is not likely to have influenced the outcome measurements, as these were objective (biomarkers of oxidative stress, inflammation and endothelial activation). Low risk of bias
Attrition bias			
**Incomplete outcome data** (Assessments should be made for each main outcome or class of outcomes)	"One of the original subjects dropped out because of personal reasons, while two were asked to discontinue participant because of a self-report of non-compliance to study requirements (p. 1089)."		Unclear risk of bias as it's not stated if these participants were or were not included in the analysis.
Reporting bias			
**Selective reporting**	**Outcomes as per methods**	**Reported in results (Yes/No)**	Low risk of bias as reported on all outcomes measured
	Oxidative stress	Yes (p. 1091)	

	Inflammation	Yes (p. 1091)	
Other bias			
**Other sources of bias**	Carry over effect: “14 days of washout between interventions (p. 1087)”		Unclear risk of bias as accounted for carry over effect and confounding but method of assessing adherence to diet is flawed as it relies on participants to recall their adherence and the study is industrially funded.
	Confounding: “Subjects were asked to maintain their weight and physical activity level as well as refrain from taking any dietary supplements or anti-inflammatory medications 2 weeks prior to and for the duration of the study.” “During the controlled feeding period of each intervention, subjects were provided with all their food. (p. 1087).”		
	Power calculation: Not stated		
	Source of funding: California Raisin Marketing Board (p. 1095).		
	Site of recruitment: Not stated		
	Adherence/compliance: Assessed by self-reported exit survey		
**Overall risk of bias**			Unclear risk of bias

**The Cochrane Collaboration's Tool for Assessing Risk of Bias**		**Study Design: Double-Blind, Randomized Cross over Trial**
**Study Details:** Auclair, S.; et al. The regular consumption of a polyphenol-rich apple does not influence endothelial function: A randomised double-blind trial in hypercholesterolemic adults *Eur. J. Clin. Nutr.* **2010**, *64*, 1158–1165.		
**Domain**	**Support for judgment**	**Review authors' judgment**
Selection bias		
**Random sequence generation**	"double-blind, randomized crossover trial"	Unclear risk of bias as method of randomization not reported
**Allocation concealment**	Not stated	Unclear risk of bias as allocation concealment not reported
Performance bias		
**Blinding of participants and personnel** (Assessments should be made for each main outcome or class of outcomes)	The study design was a double-blinded crossover. (p. 1159). The investigators were blinded with regard to the nature of the apple samples, as were the participants, This was ensured by balancing the samples for simple sugars and dietary fibres, creating homogenous samples (with exception of course to the polyphenol content)	Low risk of bias
Detection bias		
**Blinding outcome assessment** (Assessments should be made for each main outcome or class of outcomes)	"Investigators were blinded with regard to the nature of the apple sample. The study design was a double-blinded crossover." (p. 1159).	Low risk of bias
Attrition bias		
**Incomplete outcome data** (Assessments should be made for each main outcome or class of outcomes)	"A total of 30 hypercholesterolemic men … were included in the study (p. 1159)". Results section reports on baseline characteristics of 30 volunteers (p. 1160). Insufficient reporting of dropouts and no mention of missing data.	Low risk of bias as this suggests that all participants completed the study was included in analysis.
Reporting bias		
**Selective reporting**	Reported on FMD and biochemical parameters as per methods (p. 1162).	Low risk of bias as all outcomes were reported
Other bias		
**Other sources of bias**	Carry over effect: "4 week washout period" (p. 1158)	Low risk of bias due to method of reducing carry

	Confounding: "maintained their usual diet during the whole study (p. 1160)."	over effect, bias due to non-compliance and confounding from diet.
	Power calculation: Not done	
	Source of funding: "This work was supported by the European Community (p. 1163)."	
	Site of recruitment: Not stated.	
	Adherence/compliance: "Unused bags were returned at the following visit and were counted to check for compliance (pp. 1159–1160)."<br>"Compliance was assessed by measuring phloretin excretion in urine (p. 1160)"	
**Overall risk of bias**		Low risk of bias

The Cochrane Collaboration's Tool for Assessing Risk of Bias		Study Design: Randomised, Double-Blind, Placebo-Controlled Trial
**Study Details:** Wright, O.R.; Netzel, G.A.; Sakzewski, A.R. A randomized, double-blind, placebo-controlled trial of the effect of dried purple carrot on body mass, lipids, blood pressure, body composition, and inflammatory markers in overweight and obese adults: the QUENCH trial. *Can. J. Physiol. Pharmacol.* **2013**, *91*, 480–488.		
**Domain**	**Support for judgment**	**Review authors' judgment**
Selection bias		
**Random sequence generation**	States it's a randomised, double blind, placebo-controlled trial.	Unclear risk of bias as method of randomization not reported
**Allocation concealment**	Not stated	Unclear risk of bias as allocation concealment not reported
Performance bias		
**Blinding of participants and personnel** (Assessments should be made for each main outcome or class of outcomes)	"All participants and study investigators were blinded to whether participants were consuming the intervention or the placebo throughout the trial (p. 481)" "The control was dried orange carrot. 'It was coloured purple using natural purple colouring. (p. 481)"	Low risk of bias
Detection bias		
**Blinding outcome assessment** (Assessments should be made for each main outcome or class of outcomes)	States its double blinded "All participants and study investigators were blinded to whether participants were consuming the intervention or the placebo throughout the trial (p. 481)"	Low risk of bias
Attrition bias		
**Incomplete outcome data** (Assessments should be made for each main outcome or class of outcomes)	"…one not completing for unknown reasons. This participant was included in the final analysis, in line with the intention-to-treat analysis."	Low risk of bias as all participants were accounted for
Reporting bias		
**Selective reporting**	All outcomes measured were reported as evidenced by table and results section on p. 483.	Low risk of bias
Other bias		
**Other sources of bias**	Carry over effect: N/A as parallel design	Low risk of bias but likely that the

	Confounding: "Potential participants were excluded if they were already consuming purple carrots or purple carrot products (p. 481)." "The study was restricted to males to minimize confounding due to gender. Males and females are known to differ systematically for 2 of the key outcome measures of the trial: inflammatory state and body composition. Females experience regular fluctuations in hormones that influence inflammatory state, and generally have a higher proportion of body fat than males. (p. 481)." "Participants were requested to maintain their usual dietary and physical activity habits for the duration of the study. (p. 481)" "Participants completed the Wollongong Dietary Inventory to measure dietary intake at baseline and 4 weeks. Asked for brief description of the amount of time spent in intentional physical activity per week to qualitatively monitor whether this changed during the trial (p. 482)."	study is underpowered to see a significant effect
	Power calculation: No stated	
	Source of funding: University of Queensland's Early Career Researcher Fund. Summer Scholarship Program. Industry funding	
	Site of recruitment: 'Email advertisements posted by the Wesley Research Institute, Brisbane, Queensland, University of Queensland, and through a commercial television program were screened via telephone. (p. 481)"	
	Adherence/compliance: "Participants completed an intervention intake form for each day of the trial. This was cross-checked against the empty sachet packets returned at follow-up (p. 482)."	
**Overall risk of bias**		Low risk of bias

**The Cochrane Collaboration's Tool for Assessing Risk of Bias**		**Study Design: Randomised Single-Blind Placebo Controlled Parallel Design**
**Study Details:** De Maat, M.P.; Pijl, H.; Kluft, C.; Princen, H.M. Consumption of black and green tea had no effect on inflammation, haemostasis and endothelial markers in smoking healthy individuals. *Eur. J. Clin. Nutr.* **2000**, *54*, 757–763.		
**Domain**	**Support for judgment**	**Review authors' judgment**
Selection bias		
**Random sequence generation**	"Randomised study (p. 757)"	Unclear risk of bias as method of randomization not reported
**Allocation concealment**	Not stated	Unclear risk of bias as allocation concealment not stated
Performance bias		
**Blinding of participants and personnel** (Assessments should be made for each main outcome or class of outcomes)	States single-blinded but also states "control beverage was mineral water (p. 758)"	Unclear risk of bias it suggests that participants were masked but did not state method of providing mineral water appear and taste similar to intervention
Detection bias		
**Blinding outcome assessment** (Assessments should be made for each main outcome or class of outcomes)	Not stated	Low risk of bias due to objective outcomes. Study investigators are presumed to have been blinded to treatment group however it is not cleat how this was achieved.
Attrition bias		
**Incomplete outcome data** (Assessments should be made for each main outcome or class of outcomes)	"Five subjects did not complete the study (three dropped out during the run-in period and two dropped out during the intervention period), all because of social circumstances (p. 758)." results table states "for all subject" (p. 760).	Unclear risk of bias as statement in results table suggests an intention to treat analysis was performed but no mention that ITT analysis performed in text.
Reporting bias		
**Selective reporting**	All outcomes measured were reported in table on p. 760.	Low risk of bias
Other bias		
**Other sources of bias**	Carry over effect: "During a run-in period of 2 weeks the subjects drank six cups (50 mL) of the control beverage (mineral water) daily (p. 758)."	Low risk of bias

	Confounding: "The subjects were instructed by a dietitian to adhere to their normal eating habits during the intervention as closely as possible. (p. 758)"	
	Power calculation: Not stated	
	Source of funding: Unilever Research, Vaardingen, The Netherlands (p. 761)	
	Site of recruitment: "Recruited through advertisements in local newspapers and in Leiden University Medical Centre for participation in the study (p. 758)."	
	Adherence/compliance: "The subjects were asked to stick the labels of their bags of tea or capsule boxes in a daily diary as a compliance check (p. 758)."	
**Overall risk of bias**		Unclear risk of bias

**The Cochrane Collaboration's Tool for Assessing Risk of Bias**		**Study Design: A Phase II Randomised Controlled Tea Intervention Parallel Trial**
**Study Details:** Hakim, I.A.; Harris, R.B.; Brown, S.; Chow, H.H.; Wiseman, S.; Agarwal, S.; Talbot, W. Effect of increased tea consumption on oxidative DNA damage among smokers: a randomized controlled study. *J. Nutr.* **2003**, *133*, 3303s–3309s.		
**Domain**	**Support for judgment**	**Review authors' judgment**
Selection bias		
**Random sequence generation**	"Each individual was randomly assigned to drink 4 cups/d of decaffeinated green tea, decaffeinated black tea or water (p. 3304S). 'Once subjects met eligibility criteria and successfully passed the 1-mo run-in period, randomization occurred using a random-permuted block design (block size = 6). Randomization lists were prepared prior to beginning the study, with schedules separate for men and women (p. 3305S)."	Low risk of bias as method of random number generation performed
**Allocation concealment**	No stated	Unclear risk of bias as allocation concealment not reported
Performance bias		
**Blinding of participants and personnel** (Assessments should be made for each main outcome or class of outcomes)	"Because this was a study comparing the use and consumption of real foodstuffs, it was impossible to blind the intervention to either staff or subjects (p. 3304S)." Blinding of participants and investigators was not possible due to the nature of the intervention (green tea vs. black tea vs. water). Adherence to the intervention was high (95% across all groups), however consumption was higher than required in the green tea group making it likely that knowledge of treatment influenced subjects behaviours and could have influenced results.	High risk of bias due to unmasking participants and investigators from intervention products
Detection bias		

**Blinding outcome assessment** (Assessments should be made for each main outcome or class of outcomes)	"Urinary 8-OHdG: Baseline through 4-mo samples from the same individual were batched for analysis with the laboratory blinded to treatment status (p. 3305S)"	Low risk of bias
Attrition bias		
**Incomplete outcome data** (Assessments should be made for each main outcome or class of outcomes)	"143 heavy smoker recruited (p. 3304S)'. '33 men and 100 women completed the trial and were included in this analysis." 143 subjects were randomised and 133 completed the intervention. Reasons for dropout were (1) moving out of the area and (2) not having enough time. Intention-to-treat analysis was not employed however the reasons for dropout are not related to the intervention and unlikely to influence the results	Suggests no intention to treat analysis, unclear if there would be a difference due to the small number of participants excluded. Unclear risk of bias
Reporting bias		
**Selective reporting**	Reported on outcomes measured as per results section but only change from baseline and change between groups reported. No baseline and final data reported.	Low risk of bias
Other bias		
**Other sources of bias**	Carry over effect: N/A as parallel design	Low risk of bias
	Confounding: Adjusted for confounding in statistical analysis.	
	Power calculation: "A sample size of 135 individuals was estimated to provide statistical power of 80% to detect a 20% reduction in urinary excretion of 8-OHdG by either green or black tea compared with the control (water) group (p. 3306S)."	
	Source of funding: Not stated.	
	Site of recruitment: Tucson, Arizona.	

	Adherence/compliance: "Primary adherence to the study intervention was evaluated by self-reporting via monthly intake calenders. Completed 4 24 h diet assessment of maintainance of overall food intake. Short smoking questionnaire. Self-report measures of study protocol adherence and tea consumption. Measured urinary and plasma catechin levels at monthly visits (p. 3305S)."	
	Side effect monitoring: "They were telephoned during the week before each follow up visit to confirm the date and time of the next appointment and to identify any problems or side effects associated with study participation. (p. 3305S)."	
**Overall risk of bias**		Low risk of bias

**The Cochrane Collaboration's Tool for assessing risk of bias**		**Study Design: 3 Arm Randomised Cross-over Trial**
**Study Details:** Abu-Amsha Caccetta, R.; Burke, V.; Mori, T.A.; Beilin, L.J.; Puddey, I.B.; Croft, K.D. Red wine polyphenols, in the absence of alcohol, reduce lipid peroxidative stress in smoking subjects. *Free Radic. Biol. Med.* **2001**, *30*, 636–642.		
**Domain**	**Support for judgment**	**Review authors' judgment**
Selection bias		
**Random sequence generation**	"In this study using Latin Square design, volunteers were randomly allocated to drink either. (p. 637)."	Low risk of bias as random sequence generation technique used
**Allocation concealment**	Not stated	Unclear risk of bias due to allocation concealment not reported
Performance bias		
**Blinding of participants and personnel** (Assessments should be made for each main outcome or class of outcomes)	Not stated.<br>Blinding was not used in this study. Although biomarkers for compliance with alcohol consumption were measured.	Unclear risk of bias
Detection bias		
**Blinding outcome assessment** (Assessments should be made for each main outcome or class of outcomes)	Not stated.<br>Blinding was not used, however all outcome measures are objective making it unlikely that lack of blinding could have influenced the results.	Low risk of bias
Attrition bias		
**Incomplete outcome data** (Assessments should be made for each main outcome or class of outcomes)	Data reported for dealcoholised red wine group state "*n* = 17" while other groups state "*n* = 18".	Unclear risk of bias as this suggests that not all data was included or values were included in analysis or that one participant did not finish all 3 intervention periods but was included in analysis.
Reporting bias		
**Selective reporting**	**Outcomes as per methods** — **Reported in results (Yes/No)**<br>Oxidative stress — Yes (p. 639)<br>Plasma vitamins — Yes (p. 640)	Low risk of bias
Other bias		

**Other sources of bias**	Carry over effect: "a 1 week washout at the start of the study and between each beverage (p. 637)."	Unclear risk of bias<br>Carry over effect is unlikely as the investigators confirmed a 24 h return to baseline of F2-isoprostanes after alcohol consumption, meaning the 7 day washout period was sufficient.
	Confounding: "Asked to maintain smoking habits throughout the study. Subjects were instructed to always smoke the same number of cigarettes and at the same time prior to each laboratory visit. They were also asked to avoid any antioxidant supplements or over-the-counter medication and not to consume any other alcoholic beverages other than those provided (p. 637)."	
	Power calculation: Not reported	
	Source of funding: "Supported by the Australian Grape Wine Research and Development Corporation and the Medical Research Foundation of Royal Perth Hospital (p. 641)."	
	Site of recruitment: "Were recruited by advertisement from the general population (p. 637)."	
	Adherence/compliance	
**Overall risk of bias**		Unclear risk of bias

**The Cochrane Collaboration's Tool for Assessing Risk of Bias**			**Study Design: Double-Blind, Randomised, Cross over Dietary Intervention Study**
**Study Details:** Moreno-Luna, R.; Munoz-Hernandez, R.; Miranda, M.L.; Costa, A.F.; Jimenez-Jimenez, L.; Vallejo-Vaz, A.J.; Muriana, F.J.; Villar, J.; Stiefel, P. Olive oil polyphenols decrease blood pressure and improve endothelial function in young women with mild hypertension. *Am. J. Hypertens.* **2012**, *25*, 1299–1304.			
**Domain**	**Support for judgment**		**Review authors' judgment**
Selection bias			
**Random sequence generation**	"For randomization, we used a random number generation method."		Low risk of bias due to method used
**Allocation concealment**	Not stated		Unclear risk of bias as allocation concealment not reported
Performance bias			
**Blinding of participants and personnel** (Assessments should be made for each main outcome or class of outcomes)	"Despite the investigators were aware of which diet the participants received, we do not rule out the possibility that a participant could recognize the taste of virgin olive oil (p. 1300)." States double blind study (p. 1300)		Unclear risk of bias
Detection bias			
**Blinding outcome assessment** (Assessments should be made for each main outcome or class of outcomes)	Same as above		Low risk of bias
Attrition bias			
**Incomplete outcome data** (Assessments should be made for each main outcome or class of outcomes)	"Six women refused to do so, and ten more abandoned after the first dietary intervention because of protocol violation (6), intolerance to the oils (3), or change of address (1). There were 24 women completed the study. (p. 1300)." 10 more abandoned after the study. Outcome data in table states "*n* = 24" (p. 1301)		Intention- to-treat analysis may have provided indication of whether or not doing it would have affected the results. However due to cross-over design, if ITT analysis was performed it may have biased results.<br>Unclear risk of bias
Reporting bias			
**Selective reporting**	**Outcomes as per methods**	**Reported in results (Yes/No)**	Low risk of bias
	BP	Yes (p. 1301)	

	Endothelial function Yes (p. 1301)<br>Oxidative stress Yes (p. 1301)<br>Inflammation Yes (p. 1301)	
Other bias		
**Other sources of bias**	Carry over effect: "Run-inn period of 4 months (p. 1299)." "4-week washout between diets (p. 1299)."<br>Confounding: "…maintain their usual levels of exercise for the duration of the study (p. 1300)." "same calories as habitual diet (p. 1300)"<br>Power calculation: Not done<br>Source of funding: "CITOLIVA Foundation, Instituto de Salud Carlos III and Juta de Andalucia grants (p. 1303)."<br>Site of recruitment: "We consecutively asked to enter the study to forty Caucasian women that were newly diagnosed with high-normal BP or stage 1 essential hypertension (p. 1300)."<br>Adherence/compliance: "The duration of this period was to ensure adequate experience in protocol adherence (p. 1300)."	Low risk of bias
**Overall risk of bias**		Low risk of bias

**The Cochrane Collaboration's Tool for Assessing Risk of Bias**		**Study Design: Randomised Cross-over Trial**
**Study Details:** Ruano, J.; Lopez-Miranda, J.; Fuentes, F.; Moreno, J.A.; Bellido, C.; Perez-Martinez, P.; Lozano, A.; Gómez, P.; Jiménez, Y.; Jiménez, F.P. Phenolic content of virgin olive oil improves ischemic reactive hyperemia in hypercholesterolemic patients. *J. Am. Coll. Cardiol.* **2005**, *46*, 1864–1868.		
**Domain**	**Support for judgment**	**Review authors' judgment**
Selection bias		
**Random sequence generation**	"…randomised sequential crossover design. (p. 1864)"	Unclear risk of bias as method not reported
**Allocation concealment**	Comment: Not stated	Unclear risk of bias as not reported
Performance bias		
**Blinding of participants and personnel** (Assessments should be made for each main outcome or class of outcomes)	Comment: Not stated	Suggests that participants were not masked<br>High risk of bias<br>Unclear risk of bias
Detection bias		
**Blinding outcome assessment** (Assessments should be made for each main outcome or class of outcomes)	Comment: Not stated	Suggests that investigators were not masked<br>High risk of bias<br>Low risk of bias due to objective measures
Attrition bias		
**Incomplete outcome data** (Assessments should be made for each main outcome or class of outcomes)	Comment: No dropouts stated.	Suggests that all participants were included in the analysis but unclear risk of bias
Reporting bias		
**Selective reporting**	Comment: All outcomes measured were reported. **Basal lipid parameters were not shown but are not found to be significantly differently between participants in either group.	Low risk of bias
Other bias		
**Other sources of bias**	Carry over effect: No washout period stated.<br>Confounding: Not stated .No dietary assessment done.<br>Power calculation: Not stated<br>Source of funding: Not stated<br>Site of recruitment: "from the Lipids and Stheroscleosis Unit at Hospital Univeritario Reina Sofia (Cordoba, Spain) participated in the study (p. 1864)."<br>Adherence/compliance: N/A as administered once by investigator	High risk of bias as design do not account for carry over effect.
**Overall risk of bias**		Unclear risk of bias

**The Cochrane Collaboration's Tool for Assessing Risk of Bias**		**Study Design: Double-Blind Randomised Trial**
**Study Details:** Widmer, R.J.; Freund, M.A.; Flammer, A.J.; Sexton, J.; Lennon, R.; Romani, A.; Mulinacci, N.; Vinceri, F.F.; Lerman, L.O.; Lerman, A. Beneficial effects of polyphenol-rich olive oil in patients with early atherosclerosis. *Eur. J. Nutr.* **2013**, *52*, 1223–1231.		
**Domain**	**Support for judgment**	**Review authors' judgment**
Selection bias		
**Random sequence generation**	Participants were then randomised to receive a once daily serving of 30ml of either EGCG containing OO or OO alone for a total duration of four months (p. 3)	Unclear risk of bias as method of randomization not reported
**Allocation concealment**	Not stated	Unclear risk of bias as allocation concealment not reported
Performance bias		
**Blinding of participants and personnel** (Assessments should be made for each main outcome or class of outcomes)	States it's a double blinded study	Unclear risk of bias method of masking was not reported and thus cannot determine if masking can be broken
Detection bias		
**Blinding outcome assessment** (Assessments should be made for each main outcome or class of outcomes)	States it's a double blinded study	Unclear risk of bias method of masking was not reported and thus cannot determine if masking can be broken. Low risk of bias as objective measures
Attrition bias		
**Incomplete outcome data** (Assessments should be made for each main outcome or class of outcomes)	"Statistical analysis was performed by an independent statistician blinded to the randomization after completion of the studies. (p. 4)"	Tables suggest intention to treat analysis done as not all have $n = 52$, however dropouts also not stated. Suggests unclear risk of bias.
Reporting bias		
**Selective reporting**	Outcomes for within group OO-ECG for inflammatory markers not reported.	Unclear risk of bias
Other bias		
**Other sources of bias**	Carry over effect: N/A due to parallel study design.	Low risk of bias

	Confounding: "Participants were instructed to not change their diets despite olive oil supplementation, and were not given any special dietary instruction so as to have olive oil as the sole added variable in their diet (p. 3)."	
	Power calculation: Not stated	
	Source of funding: This was partly supported by Olivi Agri Team Srl-Groseeto, Italy and the University of Florence. However, the study was investigator initiated and investigator driven (p. 7).	
	Site of recruitment: 'Patients recruited from the Division of Cardiovascular Diseases at Mayo Clinic in Rochester, MN as well as by intra-institutional advertising seeking research participants. (p. 2)	
	Adherence/compliance: As above	
	Side effects: "Participants were also contacted by phone at one and three months to assess compliance and any changes in medications or symptoms (p. 3)."	
**Overall risk of bias**		Unclear risk of bias

**The Cochrane Collaboration's Tool for Assessing Risk of Bias**		**Study Design: Randomised Controlled Double-Blind Cross over Study**
**Study Details:** Clerici, C.; Nardi, E.; Battezzati, P.M.; Asciutti, S.; Castellani, D.; Corazzi, N.; Giuliano, V.; Gizzi, S.; Perriello, G.; Matteo, G.; Galli, F.; Setchell, K.D. Novel soy germ pasta improves endothelial function, blood pressure, and oxidative stress in patients with type 2 diabetes. *Diabetes Care* **2011**, *34*, 1946–1948.		
**Domain**	**Support for judgment**	**Review authors' judgment**
Selection bias		
**Random sequence generation**	"Patients were randomised to two groups. (p. 1946)."	Unclear risk of bias as method of randomization not reported
**Allocation concealment**	Not stated	Unclear risk of bias as allocation concealment not reported
Performance bias		
**Blinding of participants and personnel** (Assessments should be made for each main outcome or class of outcomes)	"…(Pasta +) and conventional pasta (Pasta-), with both packaged identically. (p. 1946)."	Suggests low risk of bias due to identical presentation. Taste may differ though
Detection bias		
**Blinding outcome assessment** (Assessments should be made for each main outcome or class of outcomes)	States "double blinded"	Unclear risk of bias as method outcomes blinded to and method were not stated<br>Low risk of bias
Attrition bias		
**Incomplete outcome data** (Assessments should be made for each main outcome or class of outcomes)	"Of the 26 patients enrolled, 6 were withdrawn (4 whose drug therapies were altered, 1 who took antioxidants, and 1 who was noncompliant to the diets) (pp. 1946–194–)." As evidenced by Supplementary Table 1: "n = 20" for oxidized LDL, 8-iso-PGF2α, GSH and IL-6, "only data concerning Period 1 were considered due to presence of sequence effect."	Low risk of bias<br>Unclear risk of bias due to reason why noncompliant participant wasn't included in analysis due to cross over design
Reporting bias		
**Selective reporting**	All outcomes measured were reported	Low risk of bias
Other bias		
**Other sources of bias**	Carry over effect: "within a 4 week washout between (p. 1946)."	Unclear risk of bias

	Confounding: Not stated	Effect seen was smaller than anticipated in power calculation suggesting risk of type 1 error is at 0.025 and study to have inadequate power.
	Power calculation: Need at least 20 subjects to observe an improvement in serum total cholesterol of about 18 mg/dL with SD = 31 mg/dL when administered enriched pasta compared with conventional pasta (Supplementary Data).	
	Source of funding: Not stated	
	Site of recruitment: Not stated.	
	Adherence/compliance: Not stated	
**Overall risk of bias**		Unclear risk of bias

**The Cochrane Collaboration's Tool for Assessing Risk of Bias**		**Study Design: Randomised Controlled Parallel-Design Trial**
**Study Details:** Yang, X.; et al. The effects of a lupin-enriched diet on oxidative stress and factors influencing vascular function in overweight subjects. *Antioxid. Redox Signal.* **2010**, *13*, 1517–1524.		
**Domain**	**Support for judgment**	**Review authors' judgment**
Selection bias		
**Random sequence generation**	"Randomisation was performed using computer-generated random numbers concealed in opaque envelopes (p. 1518)"	Low risk of bias
**Allocation concealment**	Not stated whether envelopes were sealed or not.	Unclear risk of bias
Performance bias		
**Blinding of participants and personnel** (Assessments should be made for each main outcome or class of outcomes)	Not stated.<br>Blinding is not utilised in this study. IT is unclear whther participants knew of their treatment allocation or whether there were detectable differences in terms of appearance and taste of the two treatments.	Unclear risk of bias
Detection bias		
**Blinding outcome assessment** (Assessments should be made for each main outcome or class of outcomes)	Not stated<br>Lack of blinding would be unlikely to influence the results due to the objective nature of all outcome measures.	Low risk of bias
Attrition bias		

**Incomplete outcome data** (Assessments should be made for each main outcome or class of outcomes)	Comment: no intention to treat analysis but groups in similar. 88 participants initially randomised, 14 withdrew (8due to inability to eat required amount of bread, 4 due to time restraints, 1 due to moving interstate, 1 due to change in medication). The number of dropouts appears to be even across both groups (*n* = 37 for both intervention and control groups), although the reasons for dropout may not have been similar for both treatment groups. All data for the 74 completing participants has been included.		Unclear risk of bias
Reporting bias			
**Selective reporting**	**Outcomes as per methods**	**Reported in results (Yes/No)**	Low risk of bias
	Vascular function	Yes (p. 1521)	
	Oxidative stress	Yes (p. 1521)	
Other bias			
**Other sources of bias**	Carry over effect: N/A		Risk of type 2 error due as underpowered due to inadequate sample size required to see change in outcome measures as predicted.<br>Unclear risk of bias
	Confounding: "Both groups required to replace approximately 15%–20% of their usual daily energy intake with bread. (p. 1518)" "Apart from this small shift in dietary intake, participants maintained their usual diet, physical activity, and medication regimen throughout the trial. (p. 1518)"		
	Power calculation: Based on 40 participants per group, the study was powered at 80% to detect a 25% difference in plasma and urinary<br>F2-isoprostanes and a 40% difference in plasma nitrite concentrations<br>(p. 1519). Under.		
	Source of funding: Western Australia Government (p. 1522)		
	Site of recruitment: Not stated		
	Adherence/compliance: "Compliance with the bread intake was assessed using a daily bread intake record where participants recorded the number of slices consumed each day throughout the study (p. 1518)"		
**Overall risk of bias**			Unclear risk of bias
